# Identification of New Genes Involved in Human Adipogenesis and Fat Storage

**DOI:** 10.1371/journal.pone.0031193

**Published:** 2012-02-27

**Authors:** Jörn Söhle, Nikolaus Machuy, Elma Smailbegovic, Ursula Holtzmann, Elke Grönniger, Horst Wenck, Franz Stäb, Marc Winnefeld

**Affiliations:** 1 Beiersdorf AG, Research & Development, Hamburg, Germany; 2 Max Planck Institute for Infection Biology, Molecular Biology Department, Berlin, Germany; I2MC INSERM UMR U1048, France

## Abstract

Since the worldwide increase in obesity represents a growing challenge for health care systems, new approaches are needed to effectively treat obesity and its associated diseases. One prerequisite for advances in this field is the identification of genes involved in adipogenesis and/or lipid storage. To provide a systematic analysis of genes that regulate adipose tissue biology and to establish a target-oriented compound screening, we performed a high throughput siRNA screen with primary (pre)adipocytes, using a druggable siRNA library targeting 7,784 human genes. The primary screen showed that 459 genes affected adipogenesis and/or lipid accumulation after knock-down. Out of these hits, 333 could be validated in a secondary screen using independent siRNAs and 110 genes were further regulated on the gene expression level during adipogenesis. Assuming that these genes are involved in neutral lipid storage and/or adipocyte differentiation, we performed InCell-Western analysis for the most striking hits to distinguish between the two phenotypes. Beside well known regulators of adipogenesis and neutral lipid storage (i.e. *PPARγ*, *RXR*, *Perilipin A*) the screening revealed a large number of genes which have not been previously described in the context of fatty tissue biology such as axonemal dyneins. Five out of ten axonemal dyneins were identified in our screen and quantitative RT-PCR-analysis revealed that these genes are expressed in preadipocytes and/or maturing adipocytes. Finally, to show that the genes identified in our screen are per se druggable we performed a proof of principle experiment using an antagonist for HTR2B. The results showed a very similar phenotype compared to knock-down experiments proofing the “druggability”. Thus, we identified new adipogenesis-associated genes and those involved in neutral lipid storage. Moreover, by using a druggable siRNA library the screen data provides a very attractive starting point to identify anti-obesity compounds targeting the adipose tissue.

## Introduction

Obesity is a complex medical condition affecting all populations and age groups. The rising incidence of obesity has become a global burden that is not restricted to industrialized countries [1]. According to a recent analysis conducted by the Organization for Economic Co-operation and Development (OECD), an obesity epidemic is rapidly escalating in emerging countries such as China, Mexico or Brazil [2]. Out of this reason, this global epidemic has been termed a pandemic by the World Health Organization [3]. Since obesity is related to higher rates of cardiovascular diseases, diabetes, disorders of lipid metabolism and other serious health problems, resulting social and economic consequences are multifaceted [2].

Methods for the treatment of obesity consist of dietary management, physical exercise but gastric surgical interventions and anti-obesity drugs are employed as well. Pharmacological drugs promoting weight loss work by different mechanisms of action: Sibutramine, a selective serotonin and norepinephrine reuptake inhibitor, e.g. increases the sensation of satiety [4]. The lipase inhibitor orlistat's primary function is preventing the absorption of fats in the gastrointestinal tract, thereby reducing caloric intake [4]. Currently, the role of drug treatment in obesity is limited. Since drug therapy is costly and results are often disappointing, there is a need for more effective and better tolerated treatments.

Adipose tissue regulates the body's energy balance. In periods of energy excess it provides the main energy reserve and at the same time during phases of food deprivation facilitates fat mobilization. Obesity is a disorder of energy balance and out of this reason adipose tissue is a key target to treat this condition. Due to the increase in obesity-related diseases, an enormous progress has been made determining the cellular and molecular processes underlying fat metabolism [5], [6]. Nevertheless, so far, actives which directly target the fatty tissue are rare. To overcome this limitation, new compounds have to be identified in the near future. One approach to reach this goal is to characterize new (ideally druggable) genes/gene products involved in fatty tissue biology.

RNA interference (RNAi) is a posttranscriptional silencing mechanism which is mediated through small interfering RNAs (siRNAs) [7], [8]. SiRNAs target their complementary mRNA in a sequence-specific manner, thus reducing the templates for protein synthesis. The use of the siRNA technique represents a powerful tool to study phenotypic profiles for specific knock-downs and also loss of function in cultured cells [9].

A couple of reports describe the performance of RNAi screenings to investigate the regulation of body fat storage in model organisms such as *Caenorhabditis elegans* [10] or *Drosophila* [11]. However, to our knowledge, an obesity screening in human cells has not yet been carried out. To identify new genes involved in adipogenesis and/or neutral lipid accumulation, we used a druggable siRNA-library (7,784 genes; 23,352 siRNAs) in combination with human (pre)adipocytes. Although easier and less cost-intensive we refrained from using immortalized cell lines or tumor cells since it is widely accepted that these cells may deviate from the tissue of origin. To model the *in vivo* situation as accurately as possible, we performed all our experiments with primary human cells. A druggable siRNA library was chosen, because for most genes/gene products effective compounds, regulating the action of the corresponding gene, are already available.

Our screening revealed a large number of genes which have not been previously described in the context of fatty tissue biology. Together with published data, our results offer the possibility to accelerate the development of anti-obesity treatments.

## Results

### SiRNA screening using primary human (pre)adipocytes

The aim of this study was to identify new genes influencing human adipogenesis and/or fat accumulation *in vitro*. For transfection of primary human preadipocytes, a druggable siRNA library was used containing 23,352 unique siRNAs targeting 7,784 human genes (three siRNAs per gene).

**Figure 1:**
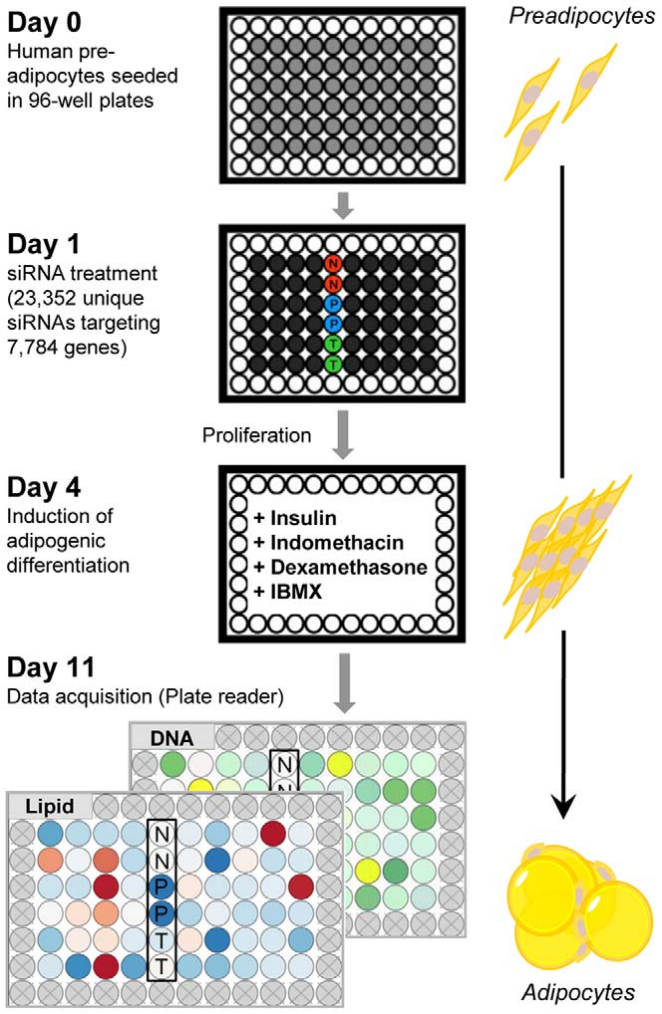
siRNA screening procedure. Two read-out-assays were performed in 96-well-plates to determine accumulation of neutral lipids and DNA-content of untransfected and transfected (pre)adipocytes (N = negative control: control (scrambled) siRNA; P = positive control, *PPARγ*-siRNA; T = transfection reagent, no siRNA). Outer wells were filled with PBS to reduce edge effects.

Successful siRNA transfection requires a cell density of approx. 30–50%, fat cell differentiation on the other hand is optimal at confluence. To align these experimental conditions, preadipocytes were siRNA transfected and then, as described earlier [12], [13], cultivated for 3 additional days allowing transfected preadipocytes to proliferate before differentiation was induced. Eleven days after cell seeding, DNA content and lipid accumulation of transfected (pre)adipocytes were determined (Figure 1). Depending on the siRNA-induced effect, changes in lipid concentration were observed.

Since *peroxisome proliferator-activated receptor γ* (*PPARγ*) is widely accepted as a master regulator of adipogenesis [14], [15], *PPARγ*-siRNA was chosen as a positive control for the primary screening. A pilot study confirmed the knock-down of *PPARγ* both on the RNA and protein level for several days after transfection using three different siRNAs (Figures S1A and B, Text S1). The knock-down efficacy correlated well with decreased neutral lipid accumulation which is a hallmark for maturing adipocytes (Figure S1C, Text S1). As negative control a scrambled-siRNA showing no homology to human genes and a siRNA-free control were utilized. For experiments, controls were performed in duplicates per plate, whereas library-associated siRNAs were arrayed in a one siRNA/one well format. The screen was carried out in a 96-well plate format to guarantee a cell density exceeding 7,500 cells which allows for a sufficient number of adipocytes to be studied. As shown in Figure 1, the outer wells were filled with phosphate buffered saline (PBS) to reduce edge-effects.

### Data correction and screen quality control

**Figure 2:**
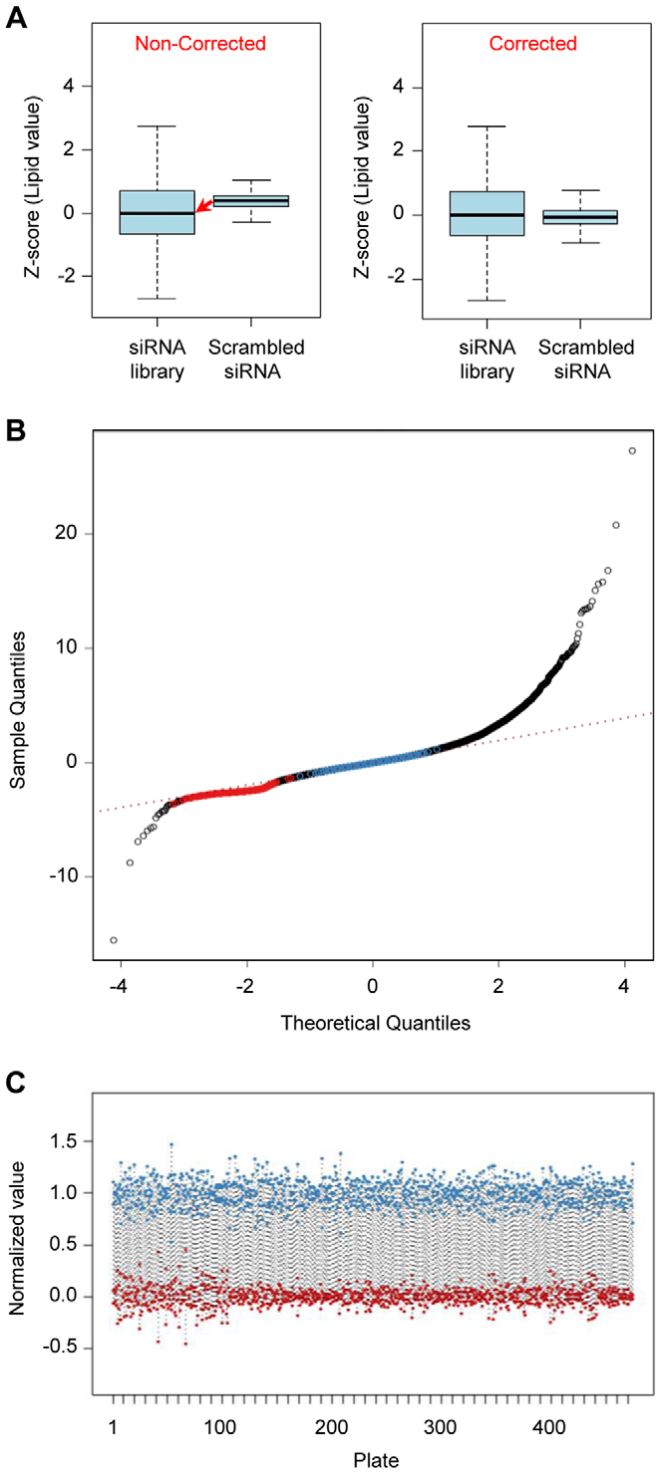
Data correction and quality control of the primary screen. (A) Correction of neutral lipid values with respect to changes in cell number. Z-scores of lipid values are illustrated before and after the amount of DNA was factored in. (B) Q-Q plot of normally distributed quantiles against screening result (Z-score) quantiles (red circles = positive control/*PPARγ*-siRNA; blue circles = negative control/scrambled siRNA). A perfect fit to a normal distribution is represented by red dotted line. (C) Experiment-wide quality plot focusing on controls. Signal from positive (red dots; *PPARγ*-siRNA) and negative (blue dots; control (scrambled) siRNA) controls plotted against plate number. The distance between the two distributions was quantified by the Z′-factor (0.42). For data normalization, the method ‘normalized percent inhibition’ (NPI) was applied.

To determine the cell number and to consider possible differences in proliferation and cell death after knock-down, a Hoechst 33342 staining was performed revealing a linear correlation between the DNA signal (Hoechst staining) and cell number (propidium iodide staining) (R^2^ = 0.973; Figure S2A, Text S1). To correct neutral lipid values for changes in cell number, the DNA signal and the signal obtained by measurement of lipids were correlated. For the primary screen and the first validation experiments cells obtained from the same donor were used and a lipid correction factor of 1.7181 was determined (R^2^ = 0.9588; N = 6, each with 8 data points; Figure S2Ba, Text S1). For a second validation experiment, using cells derived from a different donor, we calculated a correction factor of 1.522 (R^2^ = 0.9819; N = 6, each with 8 data points; Figure S2Bb). Accordingly, all neutral lipid values for the primary screen as well as for each validation experiment (first and second validation experiment = secondary screening) were adjusted using the corresponding correction factor. As depicted in Figure 2A, prior to correction of the primary screen, the mean of the Z-scores obtained for all sample lipid values slightly deviated from the Z-score determined for lipid values of the scrambled-siRNA control. After correction, the means of the Z-scores of both distributions corresponded well. In addition to the corrected Z-score values of the primary screen the quantile-quantile plot is shown in Figure 2B to visualize the distribution of each individual value. For normalization and simultaneous control of transfection efficacy, the NPI method (normalized percent inhibition) was employed. To analyze the performance of the assay during the screen, signals obtained from positive and negative controls were determined in relation to plate number. The calculated Z′ factor of 0.42 (Figure 2C) indicated an acceptable screening quality [16], especially considering that primary cells were used and cultured for 11 days before read-outs were performed.

After data correction and screen quality control, primary siRNA hits were determined. To be classified as a hit, a cutoff of a Z-score greater than 1.5 (lipid accumulation) or lower than -1.5 (lipid reduction) was selected. In addition, two out of three different siRNAs having distinct sequences had to display the same phenotype (lipid accumulation or lipid reduction). Among 7,784 tested genes, 459 genes were identified fulfilling these criteria. More precisely, 105 of these genes displayed a reduced fat accumulation during differentiation, whereas for the remaining 354 genes an increased lipid accumulation was observed.

To ensure that the observed phenotypes were caused by reduced mRNA levels after knock-down, we selected three different genes identified in our primary screen [(A) *Ephrin type-B receptor 4, EPHB4*, (B) *protein serine kinase H1, PSKH1* and (C) *v-erb-b2 erythroblastic leukemia viral oncogene homolog 2, neuro/glioblastoma derived oncogene homolog (avian), ERBB2*] and investigated knock-down efficiency. After manual siRNA transfection, we determined neutral lipid accumulation and performed qRT-PCR (Figures S3A–C, Text S1). Results illustrate that lipid accumulation correlated well with the data obtained from the primary screening and reduced mRNA levels were observed after knock-down for all genes investigated. Furthermore, no loss of viability following knock-down was detected (data not shown).

Together, these data support the assumption that the majority of observed phenotypes was caused by a knock-down of the corresponding gene rather than through off target effects.

**Figure 3:**
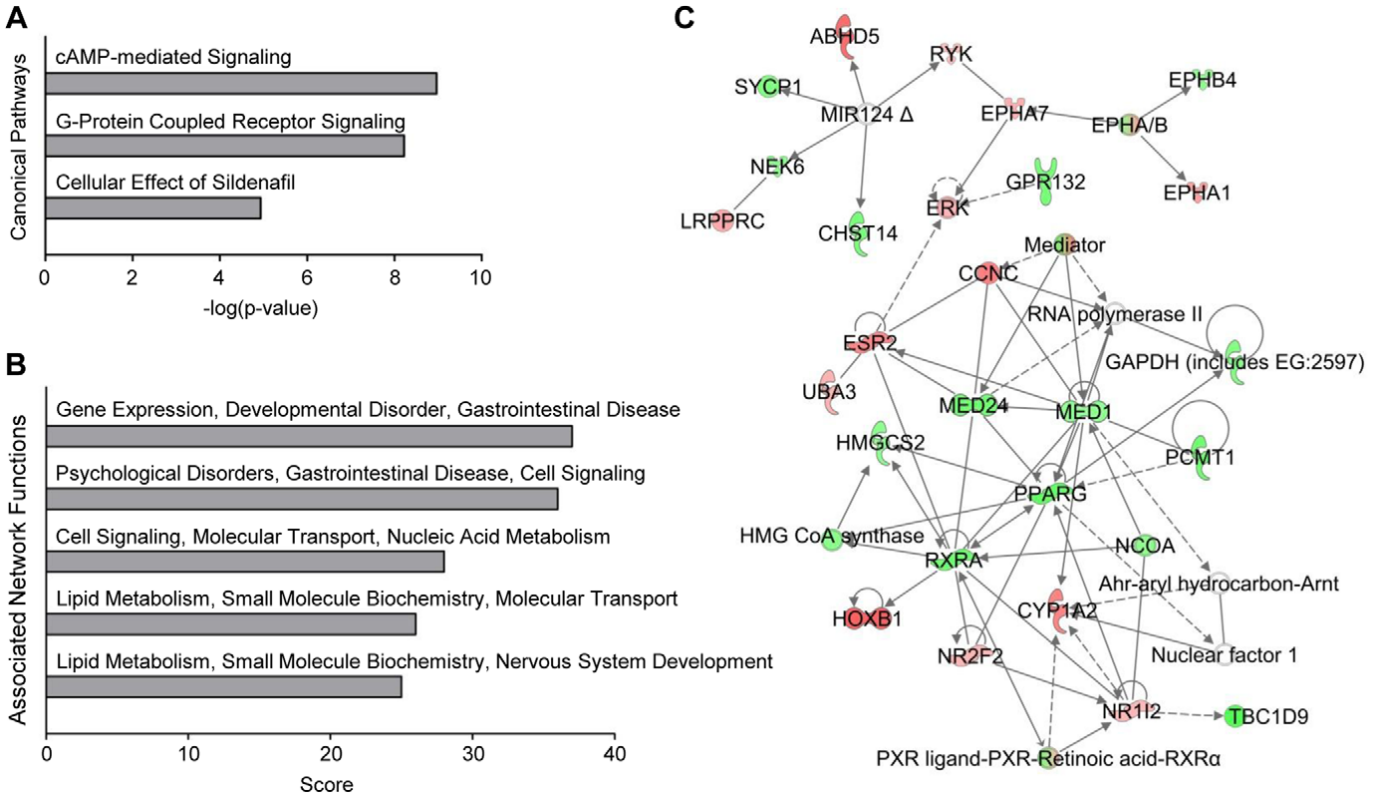
Ingenuity Pathways Analysis of positive hits identified in the primary screen. (A) Depicted are the three most significant canonical pathways with cAMP signaling being the most prominent. (B) Top five associated network functions of related genes. Two of the five top networks are believed to be directly involved in lipid metabolism. (C) The network displaying the highest score shows *PPARγ* and *RXRA*, known regulators of adipogenesis, as central genes.

To identify functional categories where the hits of the primary screen accumulated, we performed a series of pathway analyses using Ingenuity Pathways Analysis (IPA) software. For these initial analyses, we decided to use the complete set of hits identified, before performing any additional validation experiment. The rationale behind this decision was to derive more general conclusions from our results and to prevent any artificial shift of the data set caused by stringent selection criteria at this time point. As detailed in the Materials and Methods section, genes were mapped to canonical pathways revealing the following most significant categories: cAMP-mediated signaling, G-protein coupled receptor signaling, and cellular effects of sildenafil (Figure 3A). Associated network functions of related genes are depicted in Figure 3B. Among the top 5 networks, two are believed to be directly involved in lipid metabolism. The network displaying the highest score (gene expression, developmental disorder, gastrointestinal disease; score = 37; Figure 3C) shows *PPARγ* – a master regulator of adipogenesis – as a central gene.

Taken together, the results of the pathway analysis showed that a variety of proteins known to play a role in adipogenesis and fat cell metabolism were recognized in the primary screen, increasing the probability, that genes that have not yet been associated with adipogenesis and/or fat storage can be identified by this approach.

### Secondary screen

To additionally verify the results of the primary screen, we retested the 459 identified hits in two independent validation experiments using new siRNA-sequences and primary cells obtained from two different donors. In the course of our validation experiments, we observed for 333 genes (at least for one out of three newly tested siRNAs) a phenotype which resembled the one observed in the primary screen indicating that the false-positive rate was rather low.

### Characterization of identified hits

#### Axonemal dyneins are involved in fatty tissue biology

**Figure 4:**
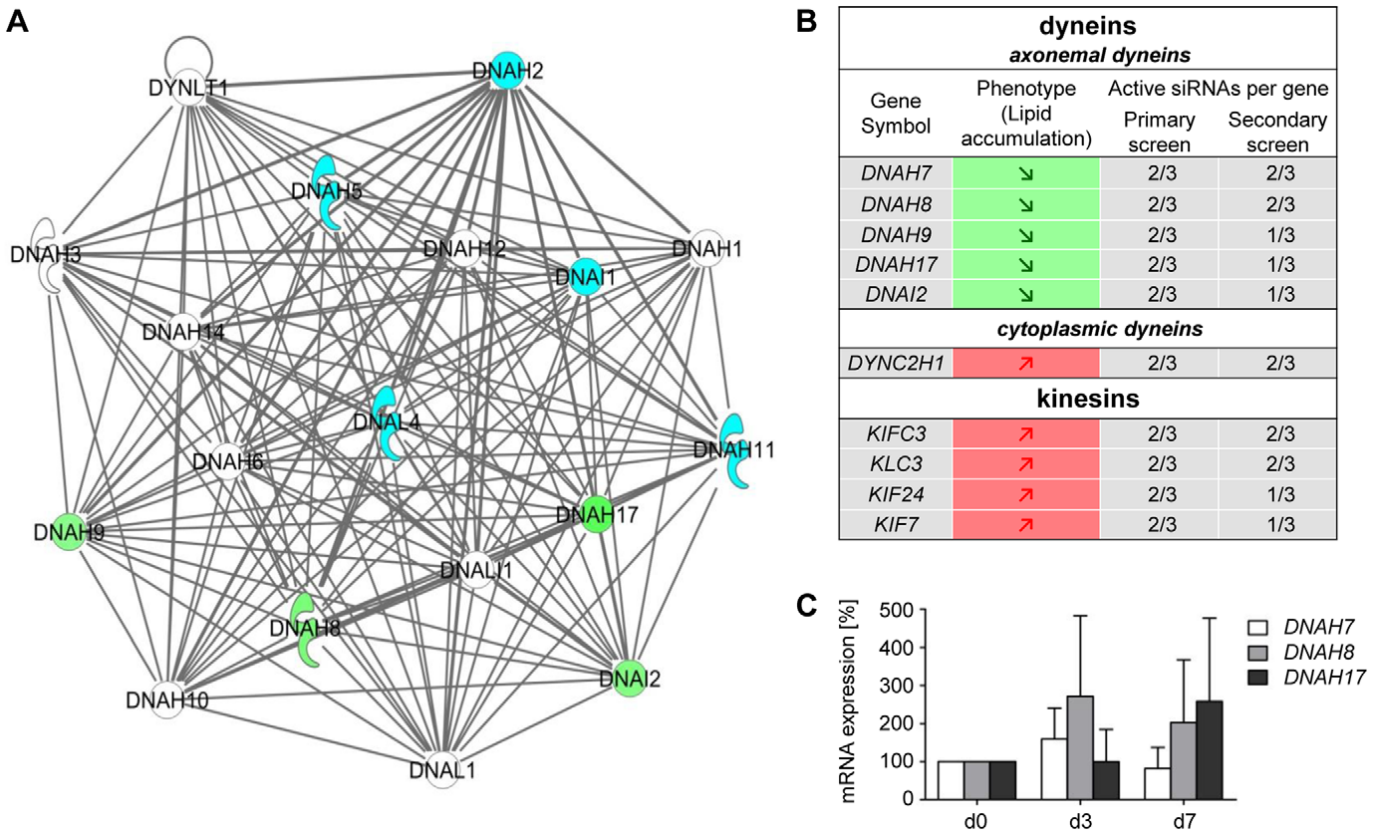
Network of axonemal dyneins. (A) Dynein network: The green coloring indicates 4 out of 5 hits regarding axonemal dyneins. *DNAH7* (also identified in our screen) was not part of the network. Blue symbols represent dyneins which were part of the library but were not determined as hits. Dyneins colored in white could not be investigated using the druggable siRNA library. (B) IPA showed an accumulation of motor proteins including dyneins and kinesins. (C) Messenger RNA levels of *DNAH7*, *DNAH8* and *DNAH17* in the course of adipocyte differentiation. Expression data at day 0 (preadipocytes) were set as 100%. All CT-values analyzed were between 28 and 34.

To characterize the 333 genes in more detail we performed – similar to the primary screen – pathway analysis using the Ingenuity software. One of the top scoring IPA generated networks showed a noticeable accumulation of axonemal dyneins (Figures 4A). Following knock-down of the human axonemal dyneins *DNAH7* (*dynein axonemal heavy chain 7*), *DNAH17* (*dynein axonemal heavy chain 17*), *DNAH8*, (*dynein axonemal heavy chain 8*), *DNAH9* (*dynein axonemal heavy chain 9*) and *DNAI2* (*dynein axonemal intermediate chain 2*) a reduced neutral lipid accumulation was detected. Interestingly, the knock-down of kinesins *KLC3* (*kinesin light chain 3*), *KIF7* (*kinesin family member 7*), *KIFC3* (*kinesin family member C3*) and *KIF24* (*kinesin family member 24*) resulted in an increased fat accumulation (Figure 4B). An increase in lipid accumulation was also observed after knock-down of the cytoplasmic dynein *DYNC2H1* (*dynein, cytoplasmatic 2, heavy chain1*). In addition, the knock-down of numerous microtubuli-binding or -associated proteins such as *MICAL1* (*microtubule associated monoxygenase, calponin and LIM domain containing 1*), *MAP7* (*microtubule-associated protein 7*), *MAST4* (*microtubule associated serine/threonine kinase family member 4*) or *TEKT2* (*tektin 2*) also caused a change in fat accumulation during adipogenesis.

To investigate the expression levels of axonemal dyneins during adipogenesis, we performed qRT-PCR analysis for these genes. Data showed, that axonemal dynein transcripts of *DNAH7*, *DNAH8* and *DNAH17* are clearly present in (pre)adipocytes (CT-values between 28 and 34) (Figure 4C), whereas the axonemal dyneins *DNAH9* and *DNAI2* are only slightly expressed showing CT-values between 36 and 37 (Figure S4, and Text S1). To show that DNAI2 and DNAH9 transcripts are truly expressed in preadipocytes we performed RT-PCR analysis using RNA obtained from control cells, DNAI2-siRNA and DNAH9-siRNA transfected cells, respectively. For independent primer-sets the results revealed, that in the control lanes (without knock-down) an amplification of DNAI2 and DNAH9 occurred, which was clearly reduced in the respective knock-down situation.

Next, we analyzed the siRNA knock-down efficiency for the dyneins DNAH7, DNAH8 and DNAH17 (Figure S5, Text S1). Following knock-down of DNAH7, DNAH8 and DNAH17 the amount of mRNA was only slightly reduced. This effect is at least partly due to the relatively small basal expression. However, a decreased mRNA expression after knock-down was detectable for all axonemal dyneins investigated.

**Figure 5:**
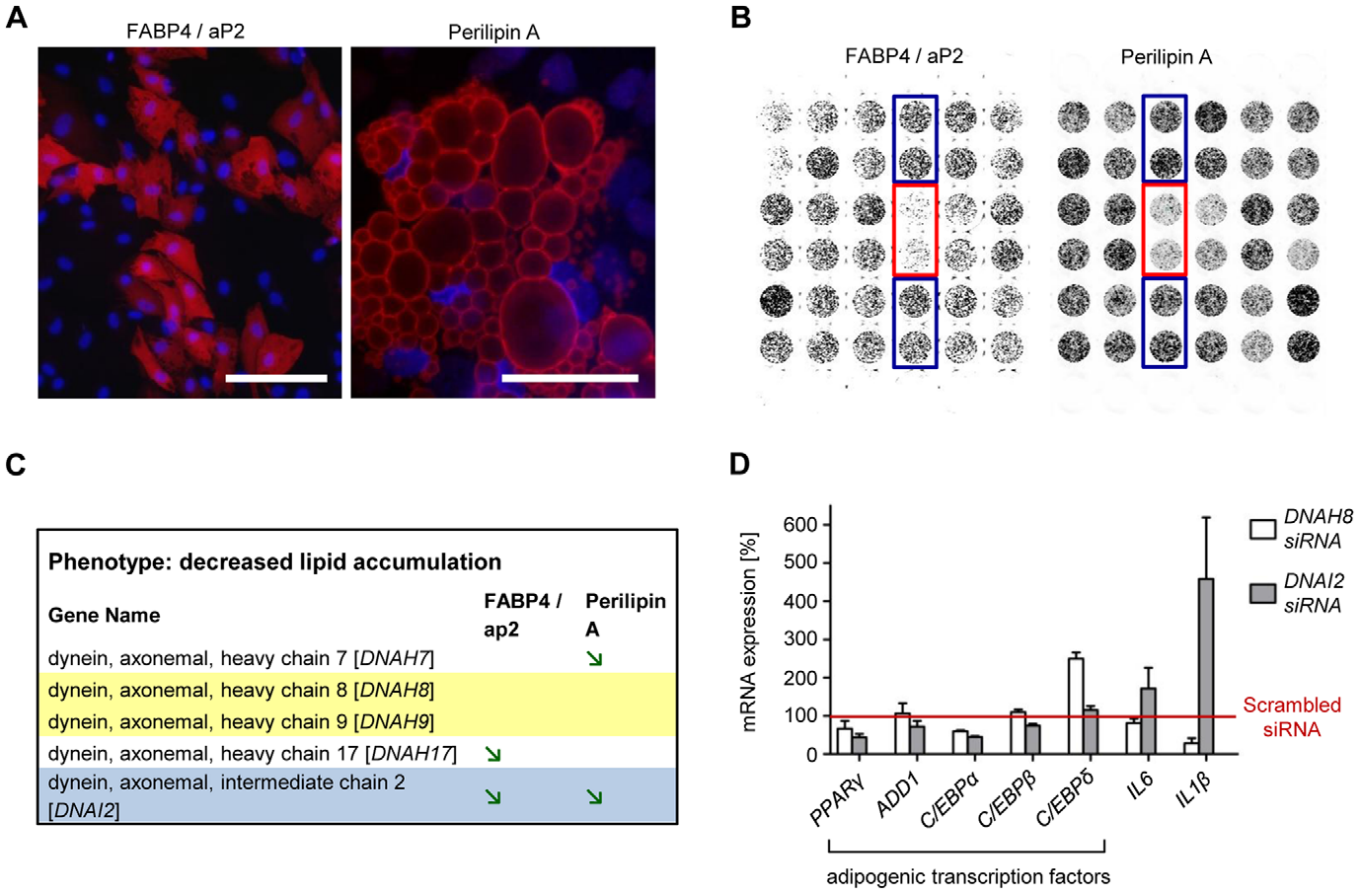
Knock-down of axonemal dyneins and classification of corresponding phenotypes. InCell-Western Analyses were performed using aP2- and Perilipin-specifc antibodies. (A) Depicted are immunofluorescence images showing aP2 and Perilipin stainings. (B) InCell-Western-Image using aP2 and Perilipin antibodies showing PPARγ knock-down (rimmed in red) and scrambled siRNA edged in blue. (C) Classify of ‘target phenotypes’ into the following categories: (1) target-knock-down that reduce differentiation, [aP2 and Perilipin A signals decreased ≥20% compared to controls, green arrow]; (2) target-knock-down that stimulate differentiation [aP2 and Perilipin A signals increased ≥20% compared to controls, red arrow] and (3) target-knock-down that caused no changes in differentiation [no significant changes in aP2 and Perilipin signals]. For targets highlighted in grey, signals of both markers decreased or increased. For those targets under laid in yellow, none marker changed. (D) Messenger RNA levels of adipocyte-relevant transcription factors as well as IL6 and IL1ß after DNAI2- and DNAH8 knock-down. Differentiation was initiated 3 days after transfection. The qRT-PCR was performed on day 5 post-transfection. Results are depicted as mean ± SD.

To study the ‘axonemal dynein knock-down phenotype’ in more detail and to determine whether a knock-down of axonemal dyneins influences the differentiation process or rather the lipid turnover, InCell-Western Analyses were carried out utilizing the adipogenic markers aP2 and Perilipin A (Figure 5A). As a reference, we used the knock-down of PPARγ. As expected, following knock-down of this master regulator of adipogenesis both adipogenic markers barely displayed a signal since cells do not differentiate into the adipogenic lineage without PPARγ being present (Figure 5B). On the basis of the results obtained from the InCell-Western Analysis, we were able to perform a first classification of target phenotypes to the following categories (Figure 5C): (1) target-knock-down that reduced differentiation [aP2 and Perilipin A signals ↓], (2) target-knock-down that stimulated differentiation [aP2 and Perilipin A signals ↑] and (3) target-knock-down that caused changes in lipid metabolism [no significant alterations in aP2 and Perilipin A signals] (for detailed information please see the Materials and Methods section). Our data show that the expression of the adipogenic markers (aP2 und Perilipin A) was strongly decreased only after knock-down of DNAI2. For DNAH7 and DNAH17 only one marker (aP2 or Perilipin A) showed expression changes according to our criteria after knock-down. This implies that at least DNAI2 (and possibly DNAH7 and DNAH17) play a role in adipogenic differentiation. In contrast, DNAH8 and DNAH9 may be involved in lipid turnover. To further characterize axonemal dyneins and their precise role in adipose tissue biology, we selected DNAI2 (“differentiation phenotype” after knock-down) and DNAH8 (“lipid turnover phenotype” after knock-down) and performed low density arrays (LDAs) after siRNA transfection. We analyzed in total 46 genes known to be involved in adipogenesis and/or lipid turnover. The results show (Fig. 5D) that in case of DNAI2 knock-down the majority of the adipogenic-relevant transcription factors are clearly decreased compared to control cells, which is in good agreement with the “differentiation phenotype” described above. Differences in mRNA levels after DNAH8- and DNAI2-knock-down were especially observed for C/EBPδ, IL6 and IL1ß. C/EBPδ is known to be involved in adipogenesis [17] but it is also described in the literature that C/EBPδ participates in the regulation of many genes associated with inflammation [18]. Therefore it can be speculated that in contrast to DNAI2, DNAH8 influences adipose tissue biology by regulating inflammatory processes. However, further analyses are needed to clarify the exact role of axonemal dyneins in adipogenesis and fat storage.

Taken together, although DNAH9 and DNAI2 are weakly expressed, the majority of axonemal dyneins are clearly present in human primary (pre)adipocytes and reduced mRNA levels could be detected after knock-down. In total, 30 axonemal dynein-specific siRNAs were tested for five targets and 17 siRNAs changed the cellular phenotype in the same manner (reduced lipid accumulation). Although we cannot completely role out off-target effects for some siRNAs targeting axonemal dyneins, it is conclusive that these proteins are involved in adipogenesis and lipid accumulation.

#### Hits that are differentially expressed during adipogenesis

To comprehensively analyze whether or not genes validated in the secondary screen (333 hits) undergo alterations in gene expression during adipogenesis, microarray analyses were performed. Accordingly, mRNA levels were determined at day 0, day 3 and day 7 after induction of differentiation. During adipogenesis, 110 among the 333 genes were regulated on the gene expression level (fold change ≥2 or ≤−2; p≤0.01). Specifically, among the phenotype characterized by a reduced fat accumulation after knock-down, 9 genes showed an increased expression, whereas 7 genes provided a reduced expression. In contrast, 40 genes derived from the second phenotype (augmented lipid accumulation after knock-down) displayed an increased and 54 a decreased expression during differentiation (Figure S6 and Text S1). For each phenotype, those genes showing the most significant expression change during adiopgenesis are depicted in Table 1 (A and B).

**Table 1:** Genes identified in the primary and validated in the secondary screen displaying a strong change in mRNA expression (determined by microarray analysis) during adipogenesis on day 3 or on day 7 compared with preadipocytes (d0).

A Phenotype: decreased lipid accumulation			
Gene Name	Accession no.	Fold change	p-value
perilipin 1 [*PLIN1*]	NM_002666	>100	<1E-45
inter-alpha (globulin) inhibitor H1 [*ITIH1*]	NM_002215	>100	<1E-45
peroxisome proliferator-activated receptor gamma [*PPARG*]	NM_138711	32.36	<1E-45
ring finger protein 150 [*RNF150*]	NM_020724	12.32	1.24E-08
distal-less homeobox 4 [*DLX4*]	NM_138281	8.65	1.45E-06
sphingosine-1-phosphate receptor 3 [*S1PR3*]	NM_005226	4.05	1.47E-11
frizzled homolog 3 (Drosophila) [*FZD3*]	NM_017412	3.77	8.60E-04
hydroxysteroid (17-beta) dehydrogenase 12 [*HSD17B12*]	NM_016142	3.73	9.77E-35
glucuronidase. beta [*GUSB*]	NM_000181	2.24	1.18E-11
multiple EGF-like-domains 6 [*MEGF6*]	NM_001409	*−81.28*	<1E-45
integrin. alpha 3 (antigen CD49C. alpha 3 subunit of VLA-3 receptor) [*ITGA3*]	NM_002204	*−13.85*	1.77E-20
v-myb myeloblastosis viral oncogene homolog (avian)-like 2 [*MYBL2*]	NM_002466	*−9.12*	7.60E-29
keratin 79 [*KRT79*]	NM_175834	*−3.64*	5.60E-04
integrin. alpha 6 [*ITGA6*]	NM_000210	*−2.86*	1.40E-04
ornithine decarboxylase antizyme 3 [*OAZ3*]	NM_016178	*−2.68*	6.75E-16
retinoblastoma 1 [*RB1*]	NM_000321	*−2.00*	3.50E-34

B Phenotype: increased lipid accumulation			
Gene Name	Accession no.	Fold change	p-value
phosphodiesterase 3B. cGMP-inhibited [*PDE3B*]	NM_000922	>100	<1E-45
stearoyl-CoA desaturase (delta-9-desaturase) [*SCD*]	NM_005063	>100	<1E-45
angiotensinogen (serpin peptidase inhibitor. clade A. member 8) [*AGT*]	NM_000029	93.86	<1E-45
RASD family. member 2 [*RASD2*]	NM_014310	87.29	<1E-45
mevalonate (diphospho) decarboxylase [*MVD*]	NM_002461	19.24	1.40E-45
dihydrolipoamide S-acetyltransferase [*DLAT*]	NM_001931	11.30	2.13E-43
phosphodiesterase 4D. cAMP-specific [*PDE4D*]	NM_006203	9.92	5.39E-37
solute carrier family 29 (nucleoside transporters). member 2 [*SLC29A2*]	NM_001532	9.89	2.36E-32
matrix Gla protein [*MGP*]	NM_000900	9.12	4.08E-23
ret proto-oncogene [*RET*]	NM_020975	8.24	7.81E-06
fibroblast growth factor 1 (acidic) [*FGF1*]	NM_000800	*−85.29*	<1E-45
regulator of G-protein signaling 4 [*RGS4*]	NM_005613	*−63.05*	<1E-45
G protein-coupled receptor 56 [*GPR56*]	NM_201525	*−51.50*	1.53E-19
RAB3B. member RAS oncogene family [*RAB3B*]	NM_002867	*−50.39*	9.16E-17
CXCR2 chemokine (C-X-C motif) receptor 2 [*IL8RB*]	NM_001557	*−26.95*	2.00E-05
histamine receptor H1 [*HRH1*]	NM_000861	*−21.27*	<1E-45
ADAM metallopeptidase with thrombospondin type 1 motif. 14 [*ADAMTS14*]	NM_139155	*−21.05*	1.85E-24
5-hydroxytryptamine (serotonin) receptor 2B [*HTR2B*]	NM_000867	*−12.25*	1.81E-11
nuclear receptor subfamily 1. group D. member 1 [*NR1D1*]	NM_021724	*−10.40*	9.42E-24
phosphatidic acid phosphatase type 2 domain containing 1A [*PPAPDC1A*]	NM_001030059	*−8.00*	8.99E-06

**Figure 6:**
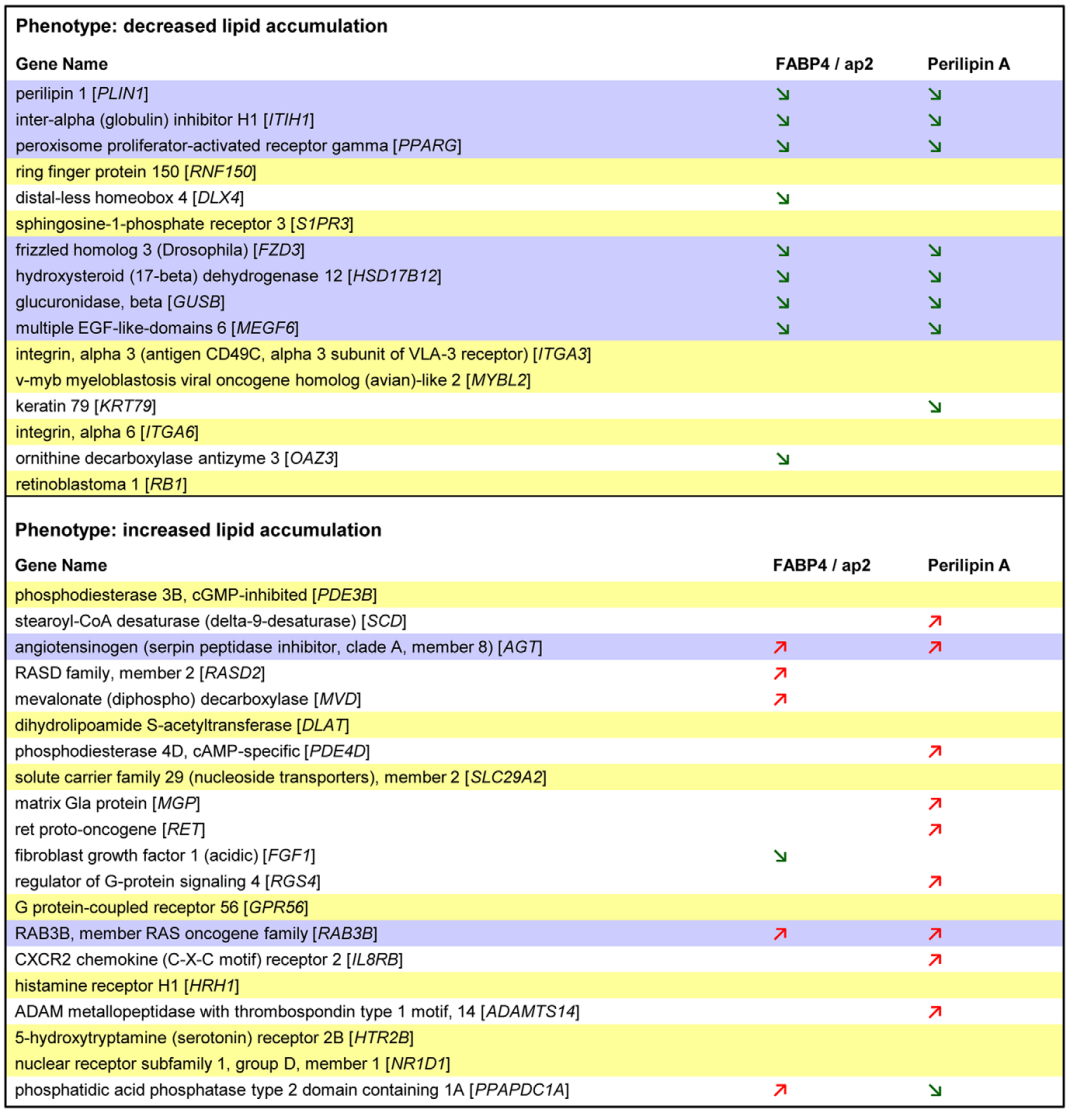
Genes identified and validated in our siRNA-screening showing the most significant changes in gene expression during adipogenesis were again transfected with specific siRNAs to classify the corresponding phenotype. Knock-down of genes caused a decreased lipid accumulation (upper box) or increased lipid accumulation (Lower box). InCell-Western Analyses were performed using aP2- and Perilipin-specific antibodies to classify target phenotypes into the following categories: (1) target-phenotype that reduce differentiation, [aP2 and Perilipin A signals decreased ≥20% compared to controls, green arrow]; (2) target-knock-down that stimulate differentiation [aP2 and Perilipin A signals increased ≥20% compared to controls, red arrow] and (3) target-phenotype that caused no changes in differentiation [none alterations in aP2 and Perilipin signals]. For targets highlighted in grey, signals of both markers decreased or increased. For those targets under laid in yellow, none marker changed.

To further analyze whether these genes (listed in Table 1) affect bona fide adipogenesis or lipid accumulation we conducted an InCell-Western Analysis as described above (for detailed information see Materials and Methods). The results indicate that for 7 genes (out of 16), showing a decreased lipid accumulation after knock-down, a reduced fat cell differentiation rate (indicated by a decreased aP2 and Perilipin A signal) could be detected. This implies that reduction in neutral lipid accumulation might rather be attributed to a decreased differentiation-efficacy than to a shift of the lipogenesis/lipolysis ratio, although an involvement of both processes cannot be completely ruled out (Figure 6, upper box, green arrows, marked in grey). For the remaining targets (except DLX4, KRT79 and OAZ3) no changes in aP2 and Perilipin A expression could be observed after knock-down. Therefore the decrease in lipid accumulation detected after knock-down of these genes might be attributed to a shift of the lipogenesis/lipolysis ratio towards lipolysis without differentiation being affected (Figure 6, upper box, no arrows, marked in yellow). It should be noted that for none of the genes showing this specific phenotype after knock-down (reduced lipid accumulation) an increase in differentiation rate could be detected (Figure 6, upper box).

The same classification was conducted for the second phenotype observed in our screen, i.e. increased lipid accumulation after knock-down. The corresponding results are illustrated in Figure 6, lower box. For two of the targets we could observe a clear increase in differentiation after knock-down (Figure 6, lower box, red arrows marked in grey) which probably caused the observed phenotype (augmented lipid accumulation). For 10 targets we detected a change in ‘differentiation’ after knock-down that was only supported by one marker (aP2 or Perilipin A) or in case of PPAPDC1A-silencing, aP2 signal increased while Perilipin A signal decreased. For this target set additional experiments are needed to distinguish between adipogenesis and/or lipid storage. For the remaining genes no changes in aP2/Perilipin A signals could be detected (Figure 6, lower box, no arrows, marked in yellow) after knock-down, which we explain by a shift of the lipogenesis/lipolysis ratio towards lipogenesis.

### Druggable targets

In our experiments we used a druggable siRNA library. For the majority of genes targeted by siRNAs of this library, compounds modulating the activity of their gene product are already available.

**Figure 7:**
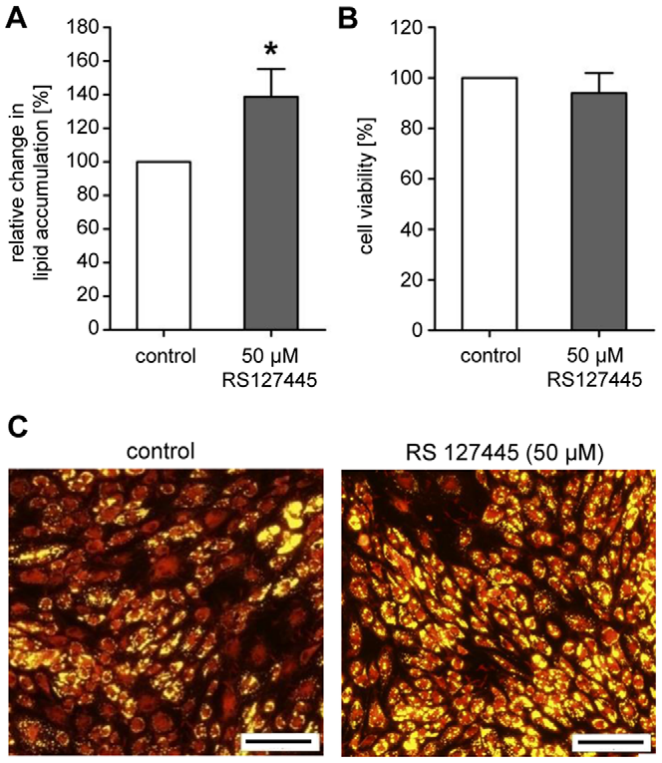
Effects of the *HTR2B*-antagonist RS127445 on neutral lipid accumulation during human primary (pre)adipocyte differentiation. (A) Increase in lipid concentration in maturing adipocytes after treatment with 50 µM RS127445. Neutral lipid accumulation is shown relative to untreated control cells set as 100%. Results are depicted as mean ± SD (n = 10). Significant differences are marked with an asterisk (* for p<0.0001). (B) Cell viability of differentiating adipocytes after treatment with 50 µM RS127445 is shown relative to untreated control cells set as 100%. Results are depicted as mean ± SD. (C) Fluorescence microscopy after incubation with or without 50 µM RS127445 and lipid staining (yellow: neutral lipids; scale bar: 200 µm).

To address the question whether or not a target-specific compound screening is per se possible, a proof of principle experiment was conducted. Therefore, we selected *HTR2B (5-hydroxytryptamine (serotonin) receptor 2B)*, a serotonin receptor which was one of our top hits identified and validated in our screening (see Table 1). Cells cultured in the presence of a commercially available antagonist of HTR2B (RS127445) exhibited significantly increased neutral lipid levels of 139 (±17)% compared to control cells (set as 100%; p≤0.0001; Figure 7A) without affecting cell viability (Figure 7B). As illustrated by the prominent yellow staining in Figure 7C, the majority of cells treated with 50 µM RS127445 accumulated neutral lipids. In contrast, control cells showed only a moderate staining. After incubation with the *HTR2B* antagonist, cells displayed a phenotype (increased fat accumulation) which had already been observed after receptor knock-down.

Taken together, these results show that a target-oriented compound screening is technically feasible. Since for most druggable gene products agonists or antagonists are already available, this approach represents a powerful tool for the development of new pharmaceuticals for a particular field of research in a shorter time period.

## Discussion

To provide a systematic analysis of genes that regulate human fat cell differentiation and/or lipid accumulation, we performed a siRNA screen using a druggable siRNA library. Although cell lines such as mouse 3T3-L1 represent a well-established model system to study fat metabolism [19], known species-related differences in adipose tissue biology and also cell line-related artifacts have to be taken into account. Out of these reasons, we utilized primary cultured human (pre)adipocytes throughout our experiments as these cells represent an *in vitro* model depicting the human situation more accurately.

In the primary screen two categories of hits were identified: those that accelerate adipogenesis and/or increase lipid accumulation and those that inhibit/delay adipogenesis and/or decrease lipid incorporation. To analyze these hits in more detail, we preformed pathway analysis. The results revealed that the highest scoring interactive network displayed *PPARγ* as a central gene. Together with its co-receptor *RXR* (*retinoic X receptor*), which was also identified in our primary screen, *PPARγ* forms heterodimers to bind DNA and activate transcription of adipocyte relevant genes to induce fat cell differentiation [5], [6], [20], [21]. In addition to PPARγ and its interacting proteins a variety of well known genes involved in adipose tissue biology (like Perilipin A etc.) were identified in our initial screen supporting the idea, that the primary screen produced reliable results. To further characterize and validate the identified hits we performed a secondary screen using independent siRNAs and cells from a different donor. The results showed that more than 70% of the candidate hits (333/459 genes) could be validated at least with one new siRNA showing that the false-positive rate was rather low.

Besides the well known players in fatty tissue biology, our screen also revealed a vast array of targets which so far have not been implicated in adipogenesis and/or lipid metabolism. Among these genes are motor proteins. Whereas cytoplasmatic dyneins and kinesins are already described in the literature to be involved in lipid-droplet transport along microtubules [22]–[25], axonemal dyneins have to our knowledge not previously been implicated in the context of adipocyte differentiation and/or neutral lipid storage. Therefore, our results show for the first time an involvement of this gene family in adipogenesis and/or lipid metabolism. Axonemal dyneins are described to be part of the so called axoneme, a structure that is found in cilia and flagella. In the axoneme these motor proteins mediate cilia or flagella movement by sliding microtubules along each other [26]. In contrast to these mobile cilia, primary “immobile” cilia exist. Many vertebrate cells create a single primary cilium in their life which was long ignored in the research community because it was thought to be just an artifact of evolution. More recently it was shown, that these structures could act as sensory antennae, transferring signals from the outside to the inside of a cell [27]. Primary cilia are formed in MSCs (mesenchymal stem cells) and the knock-down of *Polaris* (a protein that is localized to the basal body of a primary cilium) inhibits adipogenic and osteogenic differentiation. However, cells transfected with *Polaris*-siRNA showed a deficiency in adhesion and subsequently detached from the culture dish [28]. This phenotype was not observed in our experiments, suggesting that axonemal dyneins act through a different pathway. This assumption is further supported by the fact that usually axonemal dyneins are missing in primary cilia [29]. Future studies need to be carried out to investigate the role of axonemal dyneins in adipogenesis and/or neutral lipid metabolism in more detail.

Since we used a druggable library, the activity of most of these genes/gene products can be influenced by a (known) agonist or an antagonist. Out of this reason, these genes can be considered as potential targets for therapeutics. Conducting a proof of principle experiment, we inhibited the serotonin receptor *HTR2B* during adipogenesis using its commercially available antagonist RS127445 and investigated the effects on neutral lipid accumulation. Cells incubated in the presence of the antagonist showed an increased fat accumulation, similar to the knock-down phenotype, supporting the idea that a target-oriented compound screening is feasible. The Ingenuity Pathways Analysis software (Ingenuity Systems, Mountain View, CA, USA) or platforms such as The GeneCards Human Gene Database (Crown Human Genome Center, Department of Molecular Genetics, the Weizmann Institute of Science, Rehovot, Israel) allow a quick overview regarding available agonists and antagonists. Therefore, the results of this screen should accelerate compound identification. Moreover, the fact, that we also identified new pathways and genes involved in adipose tissue biology bears the interesting opportunity to modulate adipogenesis and/or fat cell differentiation at different levels preventing potential cellular compensatory mechanisms.

In conclusion, we identified new genes playing a role in adipogenesis and/or neutral lipid accumulation. Since we used a druggable siRNA library, results provide a resource for the development of novel anti-obesity compounds targeting the fatty tissue. In addition, results could be used to improve *ex vivo* fat cell differentiation aiming to assist in the generation of mesenchymal tissue used as a graft in severe cancer cases.

## Materials and Methods

### Cell culture

Subcutaneous human preadipocytes isolated from thighs of different healthy female subjects were obtained from Zen-bio Inc. (Research Triangle Park, NC, USA). Cells were cultured as previously described [30]. Briefly, cells were incubated in basal growth medium (PT-8200; Lonza, Verviers, Belgium) containing 10% fetal calf serum (PAA, Pasching, Austria), 2 mM L-glutamine, 100 U/ml penicilline and 100 µg/ml streptomycine (all obtained from Gibco BRL, Eggenstein, Germany). This medium is designated as culture medium. Differentiation into adipocytes was initiated by addition of 10 µg/ml insulin, 1 µM dexamethasone, 200 µM indomethacin and 500 µM isobutylmethylxanthine (PT-9500; Lonza) to the medium. Culture medium containing these ingredients is designated as differentiation medium. For our studies we used cells up to the third passage.

### SiRNA transfection

#### High-throughput siRNA screening (primary screen)

SiRNAs were loaded into multiwell cavities (3.6 µl/well; 2.5 µM) and diluted in OPTI-MEM I and GlutaMAX I medium (Gibco BRL) to a final volume of 15 µl. Following dilution of the transfection reagent Lipofectamine 2000 (Invitrogen, Karlsruhe, Germany) (0.27 µl in 15 µl OptiMEM I and GlutaMAX I), the resulting solution was incubated for at least 5 min, then combined with the siRNA solution and gently mixed. After an additional incubation for 20 min, 30 µl of the transfection reagent-siRNA complex were transferred to 96-well plates already containing adherend subcutaneous human preadipocytes (7,500 cells/well in 120 µl culture medium). Consequently, the final siRNA concentration was 60 nM.

Cells were incubated at 37°C and 5% CO_2_ for 3 days, before adipogenesis was induced by substitution of the medium with differentiation medium. After 7 additional days of incubation, the phenotype was determined. For that purpose, the total amount of accumulated lipids (see below) was determined and an indirect cell count (see below) was carried out by means of DNA staining. Multi-well pipetting was performed using the Biomek NX^P^ Laboratory Automation Workstation (Beckman Coulter, Brea, CA, USA). For transfection experiments, a siRNA library (Silencer® Human Druggable Genome siRNA Library V3, 384-well plates, Ambion, Darmstadt, Germany) containing the druggable human genome [31] with 3 different siRNAs per targeted gene was used. The entire library was screened once. The following siRNAs were used on every screening plate as controls: *PPARγ*-siRNA (Hs_PPARG_1_HP siRNA) und AllStars Negative Control siRNA (both obtained from Qiagen, Hilden, Germany).

#### Validation of identified hits (secondary screen)

The entirety of hits identified in the primary screen (459 genes) was retested using a miniature siRNA-library (3 independent siRNA-sequences per gene). For this validation, we used Silencer® Select siRNAs (Ambion) in a final concentration of 40 nM (optimal siRNA concentration was determined in pilot-experiments, data not shown). SiRNA transfection was performed according to the primary screen in the 96-well plate format using the Biomek NX^P^ Laboratory Automation Workstation. The screen utilizing the miniature library was carried out in two independent experiments and with two preadipocyte-populations isolated from two different donors. The following siRNAs were used on every screening plate as controls: *PPARγ*-siRNA (s10886) und Silencer Select Negative Control siRNA #1 (4390843; both obtained from Ambion).

### Neutral lipid and DNA staining

For the primary and secondary screen, the Biomek NX^P^ Laboratory Automation Workstation was employed to perform the staining.

Following induction of differentiation, cells were cultured for 7 days in 96-well plates. For lipid and parallel cell nuclei staining, cells were washed with 200 µl/well 1× PBS (PAA). Next, 200 µl/well 1× PBS containing 5 µg/ml Hoechst 33342 (Invitrogen) were added to the cells. After that, 5 µl AdipoRed™ (Lonza) were added to each well staining the intercellular lipid droplets. In these droplets cells store triacylglycerols, sterol esters and other neutral lipids [32]. After incubation for 30 min at 37°C and 5% CO_2_, fluorescence signals (AdipoRed: Ex 485/20, EM 575/15; Hoechst 33342: Ex 330/80, Em 470/40) were determined using the microplate reader Synergy 4 (BioTek Instruments GmbH, Bad Friedrichshall, Germany).

### qRT-PCR/Low Density Array

Total RNA was isolated using TRIzol® (Invitrogen) and subsequently treated with TURBO™ DNase (Ambion) according to the manufacturer's protocols. After reverse transcription with the High Capacity cDNA Reverse Transcription Kit (Applied Biosystems, Darmstadt, Germany), samples were analyzed by Real-Time TaqMan®-PCR using the 7900HT Fast-Real-Time PCR System (Applied Biosystem).

FAM labelled primers for the qRT-PCR (Applied Biosystems) were as follows: Single TaqMan Assays [*DNAH7* (Hs01022427_m1), *DNAH8* (Hs00241946_m1) and *DNAH17* (Hs01116044_m1)] and TaqMan Assays loaded into micro fluidic cards/Low Density Array [*PPARγ* (Hs00234592_m1), *ADD1* (Hs01088691_m1), *C/EBPα* (Hs00269972_s1), *C/EBPβ* (Hs00270923_s1), *C/EBPδ* (Hs00270931_s1), *IL6* (Hs00985639_m1) and *IL1β* (Hs00174097_m1)]. The PCR was performed as recommended by the supplier. Data were analyzed utilizing the Sequence detector version 2.3 software supplied with the 7900 Sequence Detector and RQ Manager 1.2. Quantification was achieved using the 2^−ΔΔCt^ method which calculates the relative change in gene expression of the target normalized to an endogenous reference (*GAPDH*; Hs99999905_m1).

### RNA preparation and Microarray analysis

To investigate gene expression changes during adipogenesis of genes identified in the primary screen, samples were analyzed using the Agilent 014850 Whole Human Genome Microarray 4×44K one-color chip (Agilent Technologies, Böblingen, Germany). Subcutaneous human preadipocytes were seeded into 6-well plates (300,000 cells/well). The following day, RNA was isolated from some of the samples (d0), while adipogenesis was initiated in the remaining samples by adding differentiation medium. Three (d3) and 7 days (d7) after induction of differentiation the respective cells were subjected to RNA isolation using TRIzol® (Invitrogen) and subsequently treated with TURBO™ DNase (Ambion) according to the manufacturer's protocols. In total, three different points in time during adipogenesis were investigated. This experiment was carried out with cells obtained from three different donors. Quality control of samples as well as performance and analysis of experiments was carried out by Miltenyi Biotech GmbH (Bergisch Gladbach, Germany).

RNA quality and integrity were determined using the Agilent RNA 6000 Nano Kit on the Agilent 2100 Bioanalyzer (Agilent Technologies). RNA was quantified by measuring A260 nm on the ND-1000 Spectrophotometer (NanoDrop Technologies, Wilmington, DE, USA).

Sample labeling was performed as detailed in the “One-Color Microarray-Based Gene Expression Analysis” protocol (version 5.7, part number G4140-90040). Briefly, 1 µg of each total RNA sample was used for the amplification and labeling step using the Agilent Quick Amp Labeling Kit (Agilent Technologies) in the presence of cyanine 3-CTP (Perkin Elmer, Waltham, MA, USA). Yields of cRNA and the dye-incorporation rate were measured with the ND-1000 Spectrophotometer (NanoDrop Technologies).

The hybridization procedure was performed according to the “One-Color Microarray-Based Gene Expression Analysis” protocol (version 5.7 part number G4140-90040) using the Agilent Gene Expression Hybridization Kit (Agilent Technologies). Briefly, 1.65 µg Cy3-labeled fragmented cRNA in hybridization buffer was hybridized overnight (17 h, 65°C) to Agilent Whole Human Genome Oligo Microarrays 4×44K using Agilent's recommended hybridization chamber and oven. Following hybridization, the microarrays were washed once with the Agilent Gene Expression Wash Buffer 1 for 1 min at room temperature followed by a second wash with preheated Agilent Gene Expression Wash Buffer 2 (37°C) for 1 min. The last washing step was performed with acetonitrile for 30 sec.

Fluorescence signals of the hybridized Agilent Microarrays were detected using Agilent's Microarray Scanner System G2505C with a resolution of 5 µm.

The Agilent Feature Extraction Software (FES) version 10.5.1.1 was used to read out and process the microarray image files. For determination of differential gene expression FES derived output data files were further analyzed using the Rosetta Resolver® gene expression data analysis system (Rosetta Biosoftware, Cambridge, MA, USA) [33].

The following ratios were determined: d0 vs. d3 and d0 vs. d7 (n = 3). The prerequisite for a gene to be considered as a regulated gene during adipogenesis was determined as follows: at least one probe per gene and at least one transcript variant per gene for the ratios d0 vs. d3 and d0 vs. d7 needed to show a fold change ≥2 or ≤−2 and p≤0.01.

### Microarray accession number

The MIAME compliant Microarray data discussed in this publication have been deposited in NCBI's Gene Expression Omnibus [34] and are accessible through GEO Series accession number GSE28628.

### In-Cell Western (ICW)/Immunofluorescence microscopic analysis

For In-Cell Western analysis, (pre-)adipocytes were fixed with 4% formaldehyde solution for 15 min at room temperature, washed with PBS and permeabilized with 0.2% Triton X-100 (5 min). After successive washing with PBS, fixed cells were pre-treated with PBS containing 10% donkey serum for 30 min. Cells were then incubated for 1 h with primary antibodies directed against adipogenic markers (Anti-FABP4/aP2, HPA002188 or Anti-Perilipin A, P1998; both obtained from Sigma) and GAPDH (sc-47724; Santa Cruz, Heidelberg, Germany). Cells were successively rinsed three times with PBS, and then incubated for 1 h with secondary antibodies labelled with IRDye680 respectively IRDye-800. Results were determined using the Odyssey Infrared Imager (Li-Cor Biosciences, Bad Homburg, Germany). In-Cell Western analyses were performed after knock-down experiments and after induction of differentiation. Cells were processed on day four (aP2 staining) and on day five (Perilipin staining) after induction of differentiation. For data interpretation, the signal intensities of the adipogenic markers were first normalized to the endogenous reference (GAPDH). Accordingly, the NPI method (normalized percent inhibition) was applied (non-targeting control = scrambled siRNA; inhibitor control = PPARγ siRNA). The threshold was determined as a deviation of +/−20% from the value of the ‘non-targeting control’. A hit was considered as validated provided at least two out of three siRNAs per gene exceeded or dropped below the predefined threshold value. The results were classified as follows: (1) In case that the knock-down of a target gene decreased the expression of aP2 and Perilipin A about more than 20% compared to their expression in control cells, targets were rated as causing a ‘reduced differentiation’. (2) In case that the knock-down of a target gene led to an increased expression of aP2 and Perilipin A (>20% compared to control), targets were rated as causing an ‘increased differentiation’. (3) Target-knock-down showing no significant expression changes with respect to aP2 and Perilipin A expression (<+/−20% compared to control after knock-down) were rated as ‘differentiation was not affected’. Please note, only if both markers shifted within the defined settings/criteria, the described classification had been applied.

The analysis was carried out in two independent experiments and with two preadipocyte populations isolated from two different donors. In addition to the ICW analyses, immunofluorescence microscopic analyses were performed by using secondary antibodies labelled with Cy3. Results were determined using the Fluorescence microscope IX71 in combination with the software cell∧F v. 2.4 (Olympus, Hamburg, Germany).

### Compound Treatment

(Pre)adipocytes were cultured in 96-well plates (10,000 cells/well) in differentiation medium (PGM-2, PT-8002, Lonza) containing 50 µM of the HTR2B antagonist RS127445 (2993, Tocris Bioscience, Ellisville, MO, USA) for 7 days. Afterwards lipid accumulation was determined using the AdipoRed™ assay according to the manufacturer's instruction. Fluorescence was detected at 572 nm using the microplate reader Infinite-M 200 (Tecan). A viability assay determining the endogenous esterase activity was used to evaluate possible cytotoxic effects of compound treatment (RS127445). Briefly, 7 days after induction of differentiation, cells were washed with 1× PBS and incubated for 20 min at 37°C and 5% CO_2_ with 100 µl/well of a fluorescein diacetate (FDA) (Sigma, Taufkirchen, Germany) solution (15 µg/ml FDA in 1× PBS). Fluorescence was determined at 517 nm using the microplate reader infinite® M200 (Tecan, Crailsheim, Germany).

### Data analysis (primary and secondary screen)

For an unbiased interpretation of the primary screen, a lipid *data correction factor* was determined. This step was performed to reduce the influence of plate effects and siRNA knock-down on proliferation and cell viability. Corrected lipid data were statistically evaluated using the cellHTS2 software [35] implemented in Bioconductor/R for the analysis of cell-based high-throughput RNAi screens. For this purpose, corrected raw data were normalized using the NPI method (normalized percent inhibition) in order to factor in the varying transfection efficiency in different plates. In an additional step, the Z-score-transformation was applied to assign a score to the normalized data taking into account the scattering of data points over all screen plates. Z-score-transformed data were then used for a redundant siRNA activity analysis (RSA) [36]. The RSA applies a rang-based hypergeometric distribution test for hit analysis. To be considered a hit, genes had to display a Z score of ≥1.5 or ≤−1.5 for at least two out of three screened siRNAs.

To minimize the risk of misinterpretation of gene activity for the secondary screen, we also applied the cellHTS2 software for statistical data analysis. In contrast to the primary screen, we did not perform a Z-score-transformation, NPI normalized data were directly used for the subsequent RSA. The threshold was determined as a deviation of +/−20% from the value of the ‘non-targeting control’. A hit was considered as validated provided at least one siRNA per gene exceeded or dropped below the predefined threshold value.

### Gene function and network analysis

For pathway analysis and analysis of gene functions and networks the Ingenuity Pathways Analysis (IPA) software, version 6.3 (Ingenuity® Systems, http://www.ingenuity.com) was used. Positive hits were analyzed in combination with the ranking information obtained from the previously performed RSA. IPA settings for analyses were as follows: Use of gene information without limitation through species, tissues or cell lines; for network analysis (max. 35 molecules/network), direct and indirect relations were taken into account; for analyses all available “data sources” provided by IPA were considered.

### Statistical analysis

A significance level of 0.05 (alpha) was chosen for statistical analysis, based on two-sided hypothesis testing. The following analysis for paired samples was performed: (1) Check of normal distribution of pair's differences by means of Shapiro-Wilk's test; (2) Comparison versus control by paired sample T-test.

For analysis, STATISTICA 9.1 software (StatSoft. Inc., Tulsa, OK, USA) was used.

## Supporting Information

Figure S1 **Determination of** ***PPARγ*** **knock-down efficacy and characterization of the resulting phenotype.** Human primary preadipocytes were transfected with three different *PPARγ*-siRNAs and differentiation was initiated after 3 days. (A) *PPARγ* knock-down was confirmed using qRT-PCR (5 days post-transfection) and (B) Immunoblot analysis (6 days post-transfection). (C) Effects on lipid accumulation after PPARγ knock-down was detected 10 days after siRNA transfection. Lipid accumulation is shown relative to control cells incubated with scrambled-siRNA (control siRNA) set as 100%. Results are depicted as mean ± SD (n = 6).
(TIF)

Figure S2 **Quality check of read-out methods and determination of lipid data correction factors.** (A) Scatter plot between the DNA signal and the number of cells showed a linear correlation (R^2^ = 0.973). Following Hoechst 33342 stain, DNA was determined using a plate reader. The number of cells was counted with an automated fluorescence microscope after propidium iodide staining. (B) Scatter plots between DNA and lipid signals showed a linear correlation for cells of two different donors. Six independent experiments with eight data points were prepared. (a) The slope of the regression line indicates the lipid data correction factor (1.7181) for the primary screen and the first validation experiment (part of the secondary screen). (b) The slope of the regression line indicates the lipid data correction factor (1.522) for the second validation experiment (part of the secondary screen).
(TIF)

Figure S3 **Knock-down efficacy and resulting phenotype of selected hits identified in the primary screen.** (A) *EPHB4*, (B) *PSKH1* and (C) *ERBB2*. Lipid accumulation according to the primary screen and after manual transfection determined for the three selected genes A–C. Manual siRNA transfection was performed for each selected gene using the 3 different siRNAs utilized in the primary screen. Differentiation was initiated 3 days after transfection. Results are depicted as mean ± SD (n = 6). To determine knock-down efficacy, qRT-PCR was performed on day 3 (d3) and day 5 (d3+2) post-transfection.
(TIF)

Figure S4 **Expression of DNAI2 and DNAH9 in (pre)adipocytes.** (A) CT values obtained by qRT-PCR analysis. (B) Amplification of DNAI2- and DNAH9 transcripts isolated from knock-down as well as from control transfected (scrambled siRNA) cell populations. Depicted are the PCR products, using two independent primer sets. (C) Lipid accumulation of DNAI2- and DNAH9 knock-down populations compared to controls (set as 100%).
(TIF)

Figure S5 **Knock-down efficacy and resulting phenotype after siRNA transfection using axonemal dynein-specific siRNAs.** (A) *DNAH7*, (B) *DNAH8* and (C) *DNAH17*. Lipid accumulation according to the screen and after manual transfection determined for the three selected genes A–C (n = 6). Manual siRNA transfection was performed for each selected gene using those siRNAs showing a phenotype in the primary screen. Differentiation was initiated 3 days after transfection. Results are depicted as mean ± SD. To determine knock-down efficacy, qRT-PCR was performed on day 5 (d3+2) post-transfection. (DNAH7: n = 2; DNAH8 and DNAH17: n = 3).
(TIF)

Figure S6 **Messenger RNA expression during adipogenesis of hits validated in the secondary screen.** Messenger RNA levels were determined by microarray analysis at different time points during adipogenesis (day 0, day 3 and day 7). Depicted is the number of hits displaying an increased (↑) or decreased (↓) gene expression level compared with preadipocytes (fold change ≥2 or ≤−2; p≤0.01).
(TIF)

Text S1 **Material and methods for experiments provided in the “supporting information” section.** A detailed description is given for: Cell culture and siRNA transfection, qRT-PCR, Immunoblotting, reverse transcription PCR and agarose gel electrophoresis, neutral lipid accumulation, DNA staining, microarray analysis and determination of the lipid correction factors.
(DOC)

## References

[1] Prentice AM (2006). The emerging epidemic of obesity in developing countries.. Int J Epidemiol.

[2] Cecchini M, Sassi F, Lauer JA, Lee YY, Guajardo-Barron V (2010). Tackling of unhealthy diets, physical inactivity, and obesity: health effects and cost-effectiveness.. Lancet.

[3] WHO (2000). Obesity: preventing and managing the global epidemic.. Report of a WHO Consultation, WHO Technical Report Series No. 894.

[4] Vetter ML, Faulconbridge LF, Webb VL, Wadden TA (2010). Behavioral and pharmacologic therapies for obesity.. Nat Rev Endocrinol.

[5] Rosen ED, Walkey CJ, Puigserver P, Spiegelman BM (2000). Transcriptional regulation of adipogenesis.. Genes Dev.

[6] Gregoire FM (2001). Adipocyte differentiation: From fibroblast to endocrine cell.. Exp Biol Med.

[7] Fire A, Xu S, Montgomery MK, Kostas SA, Driver SE (1998). Potent and specific genetic interference by double-stranded RNA in Caenorhabditis elegans.. Nature.

[8] Tuschl T, Borkhardt A (2002). Small interfering RNAs: a revolutionary tool for the analysis of gene function and gene therapy.. Mol Interv.

[9] Elbashir SM, Harborth J, Lendeckel W, Yalcin A, Weber K (2001). Duplexes of 21-nucleotide RNAs mediate RNA interference in cultured mammalian cells.. Nature.

[10] Ashrafi K, Chang FY, Watts JL, Fraser AG, Kamath RS (2003). Genome-wide RNAi analysis of Caenorhabditis elegans fat regulatory genes.. Nature.

[11] Pospisilik JA, Schramek D, Schnidar H, Cronin SJ, Nehme NT (2010). Drosophila genome-wide obesity screen reveals hedgehog as a determinant of brown versus white adipose cell fate.. Cell.

[12] Xu Y, Mirmalek-Sani S-H, Yang X, Zhang J, Oreffo RO (2006). The use of small interfering RNAs to inhibit adipocyte differentiation in human preadipocytes and fetal-femur-derived mesenchymal cells.. Exp Cell Res.

[13] Grönniger E, Wessel S, Kühn SC, Söhle J, Wenck H (2010). A new protocol for functional analysis of adipogenesis using reverse transfection technology and time-lapse video microscopy.. Cell Biol Int.

[14] Chawla A, Schwarz EJ, Dimaculangan DD, Lazar MA (1994). Peroxisome proliferator-activated receptor (PPAR) gamma: adipose-predominant expression and induction early in adipocyte differentiation.. Endocrinology.

[15] Tontonoz P, Hu E, Graves RA, Budavari AI, Spiegelman BM (1994). mPPAR gamma 2: tissue-specific regulator of an adipocyte enhancer.. Genes Dev.

[16] Birmingham A, Selfors LM, Forster T, Wrobel D, Kennedy CJ (2009). Statistical methods for analysis of high-throughput RNA interference screens.. Nat Methods.

[17] Payne VA, Au WS, Lowe CE, Rahman SM, Friedman JE (2009). C/EBP transcription factors regulate SREBP1c gene expression during adipogenesis.. Biochem J.

[18] Yan C, Zhu M, Staiger J, Johnson PF, Gao H (2011). C5a-regulated CCAAT/enhancer binding protein β and -δ are essential in Fcγ receptor- mediated inflammatory cytokine and chemokine production in macrophages.. J Biol Chem.

[19] Green H, Kehinde O (1975). An established preadipose cell line and its differentiation in culture. II. Factors affecting the adipose conversion.. Cell.

[20] Lehrke M, Lazar MA (2005). The many faces of PPARgamma.. Cell.

[21] Hamza MS, Pott S, Vega VB, Thomsen JS, Kandhadayar GS (2009). De-novo identification of PPARgamma/RXR binding sites and direct targets during adipogenesis.. PLoS One.

[22] Gross SP, Welte MA, Block SM, Wieschauss EF (2000). Dynein-mediated cargo transport in vivo. A switch controls travel distance.. J Cell Biol.

[23] Nagayama M, Uchida T, Gohara K (2007). Temporal and spatial variations of lipid droplets during adipocyte division and differentiation.. J Lipid Res.

[24] Shubeita GT, Tran SL, Xu J, Vershinin M, Cermelli S (2008). Consequences of motor copy number on the intracellular transport of kinesin-1-driven lipid droplets.. Cell.

[25] Welte MA (2009). Fat on the move: intracellular motion of lipid droplets.. Biochem Soc Trans.

[26] Lindemann CB, Lesich KA (2010). Flagellar and ciliary beating: the proven and the possible.. J Cell Sci.

[27] Berbari NF, O'Connor AK, Haycraft CJ, Yoder BK (2009). The primary cilium as a complex signaling center.. Curr Biol.

[28] Tummala P, Arnsdorf EJ, Jacobs CR (2010). The Role of Primary Cilia in Mesenchymal Stem Cell Differentiation: A Pivotal Switch in Guiding Lineage Commitment.. Cell Mol Bioeng.

[29] Satir P, Christensen ST (2007). Overview of structure and function of mammalian cilia.. Annu Rev Physiol.

[30] Söhle J, Knott A, Holtzmann U, Siegner R, Grönniger E (2009). White Tea extract induces lipolytic activity and inhibits adipogenesis in human subcutaneous (pre)-adipocytes.. Nutr Metab (Lond).

[31] Hopkins AL, Groom CR (2002). The druggable genome.. Nat Rev Drug Discov.

[32] Olofsson SO, Boström P, Andersson L, Rutberg M, Perman J (2009). Lipid droplets as dynamic organelles connecting storage and efflux of lipids.. J Biochim Biophys Acta.

[33] Weng L, Dai H, Zhan Y, He Y, Stepaniants SB (2006). Rosetta error model for gene expression analysis.. Bioinformatics.

[34] Edgar R, Domrachev M, Lash AE (2002). Gene Expression Omnibus: NCBI gene expression and hybridization array data repository.. Nucleic Acids Res.

[35] Boutros M, Brás LP, Huber W (2006). Analysis of cell-based RNAi screens.. Genome Biol.

[36] König R, Chiang CY, Tu BP, Yan SF, DeJesus PD (2007). A probability-based approach for the analysis of large-scale RNAi screens.. Nat Methods.

