# Supplementary figures and images for: Identification of New Genes Involved in Human Adipogenesis and Fat Storage
Source: PLoS One. 2012 Feb 27;7(2):e31193. doi: 10.1371/journal.pone.0031193 (PMC3287999; doi:10.1371/journal.pone.0031193)

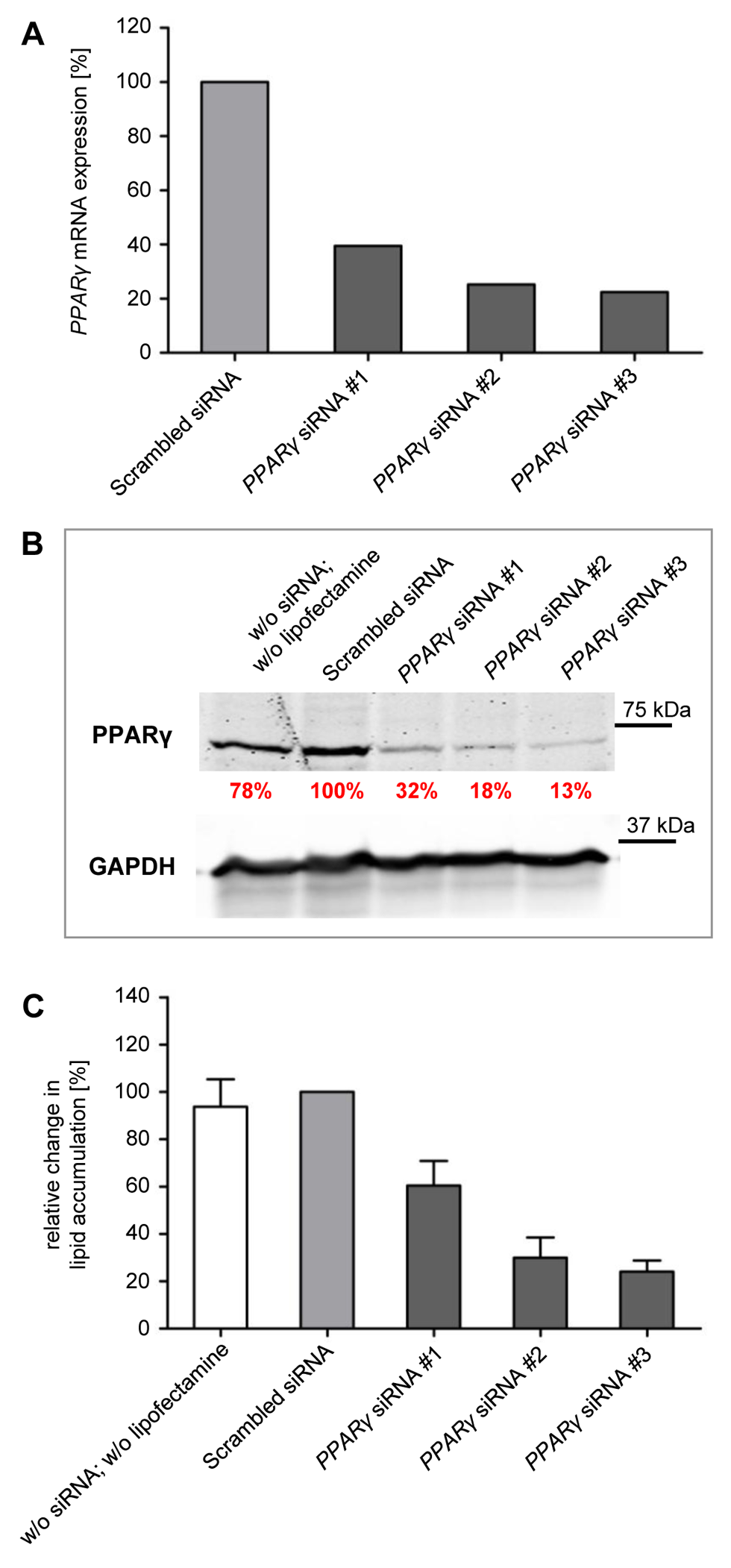

Supplement: Figure S1 — Determination of PPARγ knock-down efficacy and characterization of the resulting phenotype. Human primary preadipocytes were transfected with three different PPARγ-siRNAs and differentiation was initiated after 3 days. (A) PPARγ knock-down was confirmed using qRT-PCR (5 days post-transfection) and (B) Immunoblot analysis (6 days post-transfection). (C) Effects on lipid accumulation after PPARγ knock-down was detected 10 days after siRNA transfection. Lipid accumulation is shown relative to control cells incubated with scrambled-siRNA (control siRNA) set as 100%. Results are depicted as mean ± SD (n = 6). (TIF) [file pone.0031193.s001.tif]

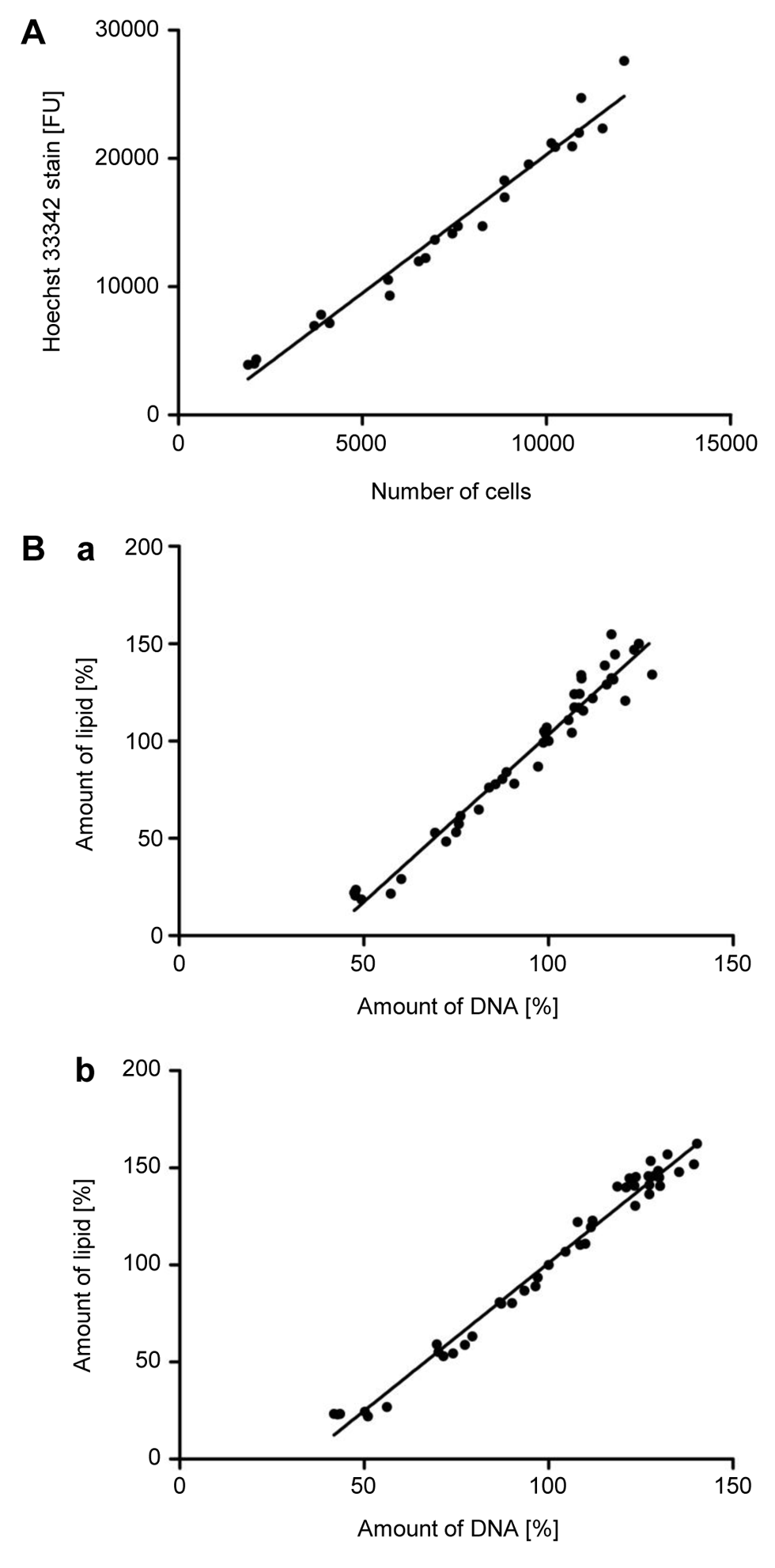

Supplement: Figure S2 — Quality check of read-out methods and determination of lipid data correction factors. (A) Scatter plot between the DNA signal and the number of cells showed a linear correlation (R2 = 0.973). Following Hoechst 33342 stain, DNA was determined using a plate reader. The number of cells was counted with an automated fluorescence microscope after propidium iodide staining. (B) Scatter plots between DNA and lipid signals showed a linear correlation for cells of two different donors. Six independent experiments with eight data points were prepared. (a) The slope of the regression line indicates the lipid data correction factor (1.7181) for the primary screen and the first validation experiment (part of the secondary screen). (b) The slope of the regression line indicates the lipid data correction factor (1.522) for the second validation experiment (part of the secondary screen). (TIF) [file pone.0031193.s002.tif]

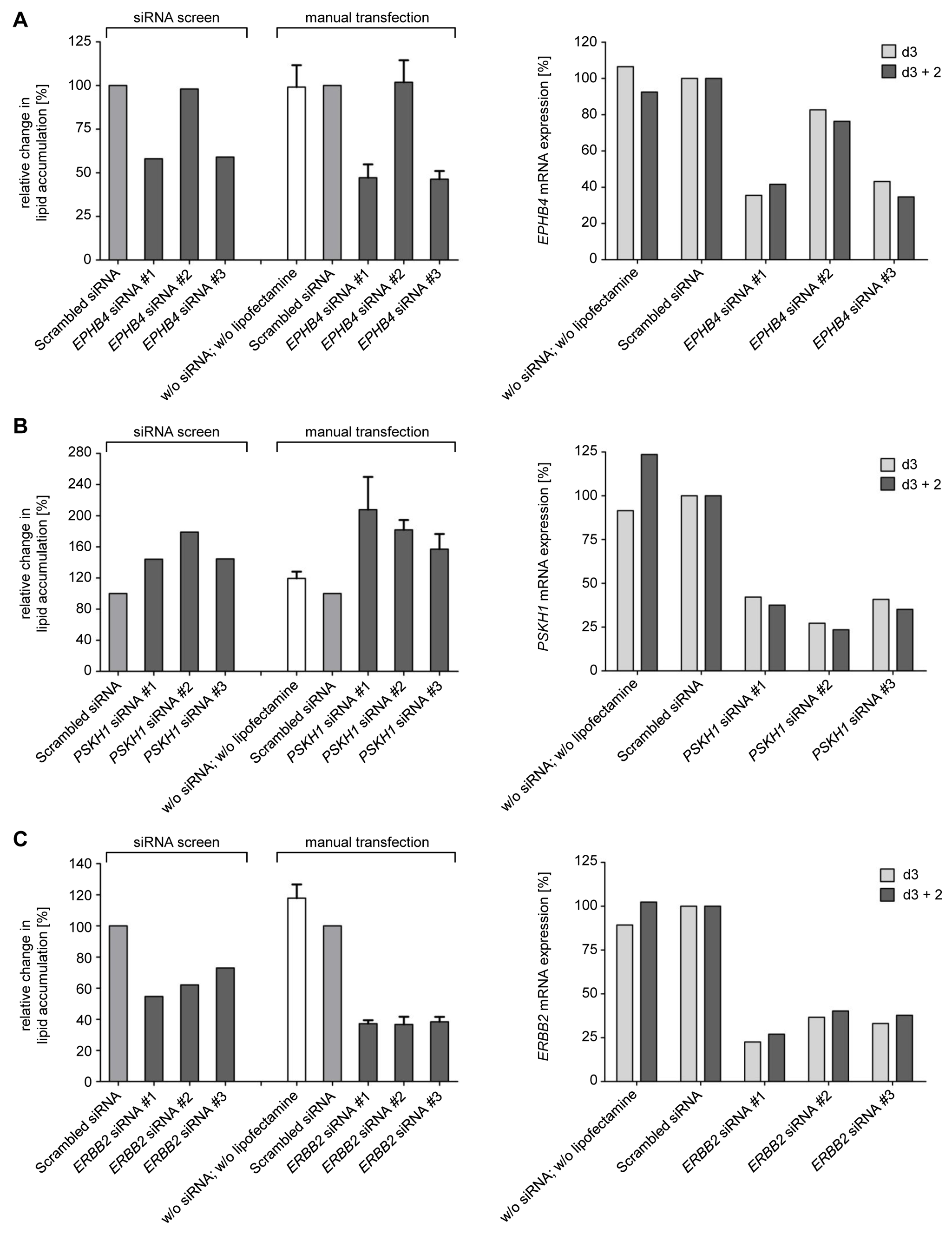

Supplement: Figure S3 — Knock-down efficacy and resulting phenotype of selected hits identified in the primary screen. (A) EPHB4, (B) PSKH1 and (C) ERBB2. Lipid accumulation according to the primary screen and after manual transfection determined for the three selected genes A–C. Manual siRNA transfection was performed for each selected gene using the 3 different siRNAs utilized in the primary screen. Differentiation was initiated 3 days after transfection. Results are depicted as mean ± SD (n = 6). To determine knock-down efficacy, qRT-PCR was performed on day 3 (d3) and day 5 (d3+2) post-transfection. (TIF) [file pone.0031193.s003.tif]

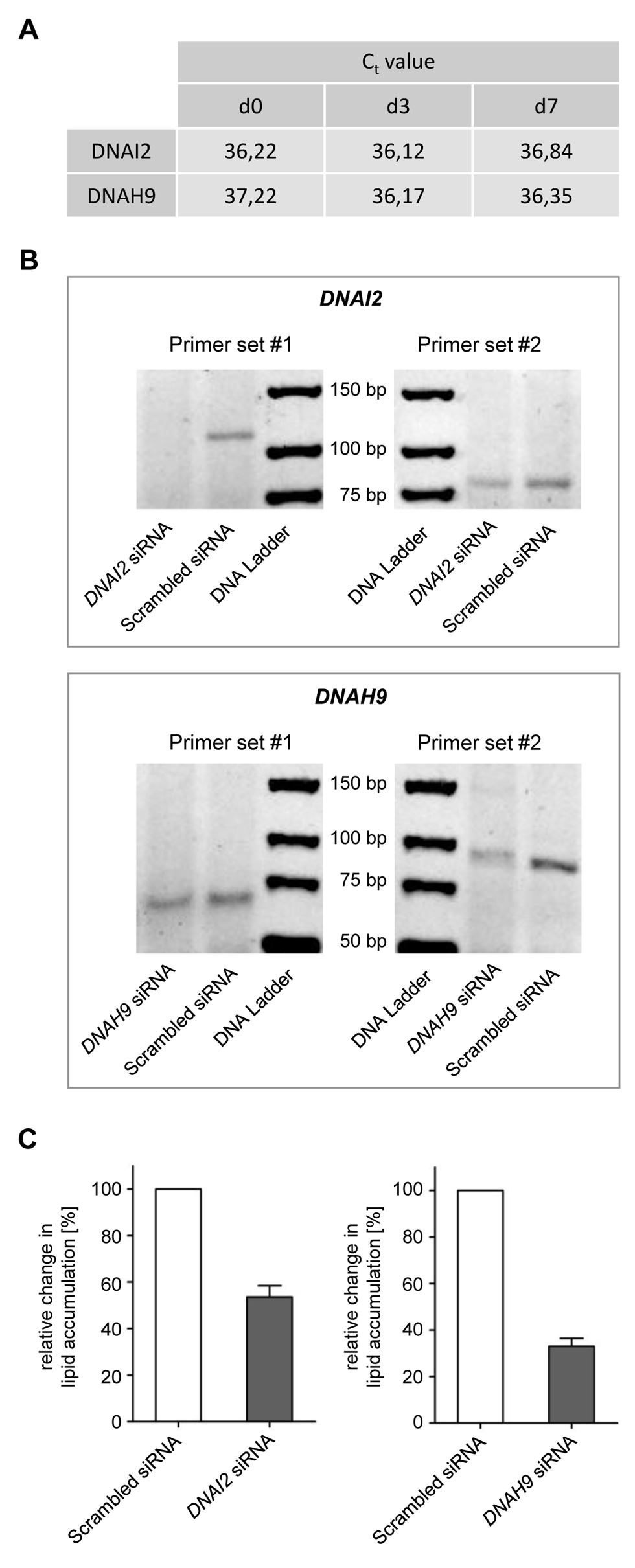

Supplement: Figure S4 — Expression of DNAI2 and DNAH9 in (pre)adipocytes. (A) CT values obtained by qRT-PCR analysis. (B) Amplification of DNAI2- and DNAH9 transcripts isolated from knock-down as well as from control transfected (scrambled siRNA) cell populations. Depicted are the PCR products, using two independent primer sets. (C) Lipid accumulation of DNAI2- and DNAH9 knock-down populations compared to controls (set as 100%). (TIF) [file pone.0031193.s004.tif]

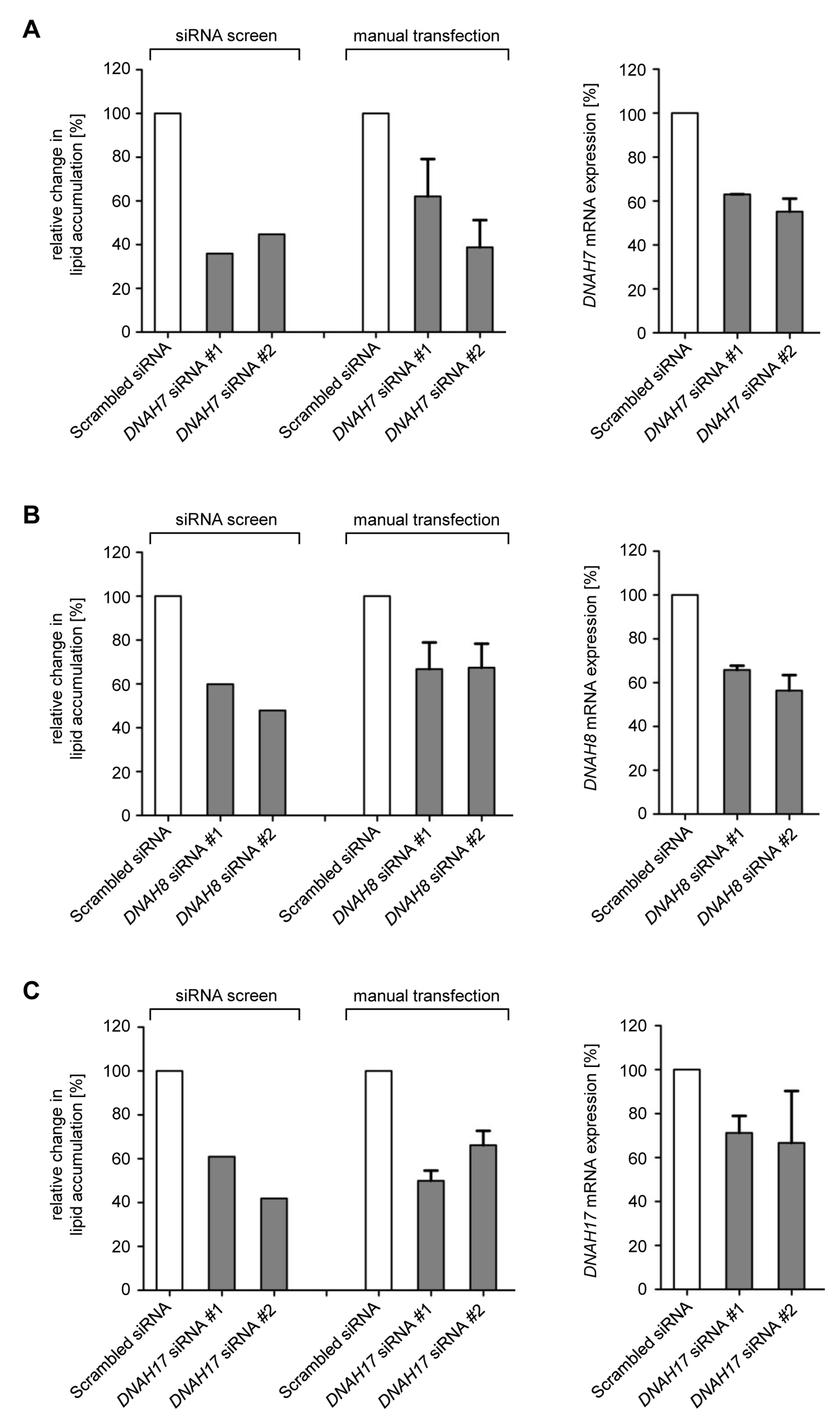

Supplement: Figure S5 — Knock-down efficacy and resulting phenotype after siRNA transfection using axonemal dynein-specific siRNAs. (A) DNAH7, (B) DNAH8 and (C) DNAH17. Lipid accumulation according to the screen and after manual transfection determined for the three selected genes A–C (n = 6). Manual siRNA transfection was performed for each selected gene using those siRNAs showing a phenotype in the primary screen. Differentiation was initiated 3 days after transfection. Results are depicted as mean ± SD. To determine knock-down efficacy, qRT-PCR was performed on day 5 (d3+2) post-transfection. (DNAH7: n = 2; DNAH8 and DNAH17: n = 3). (TIF) [file pone.0031193.s005.tif]

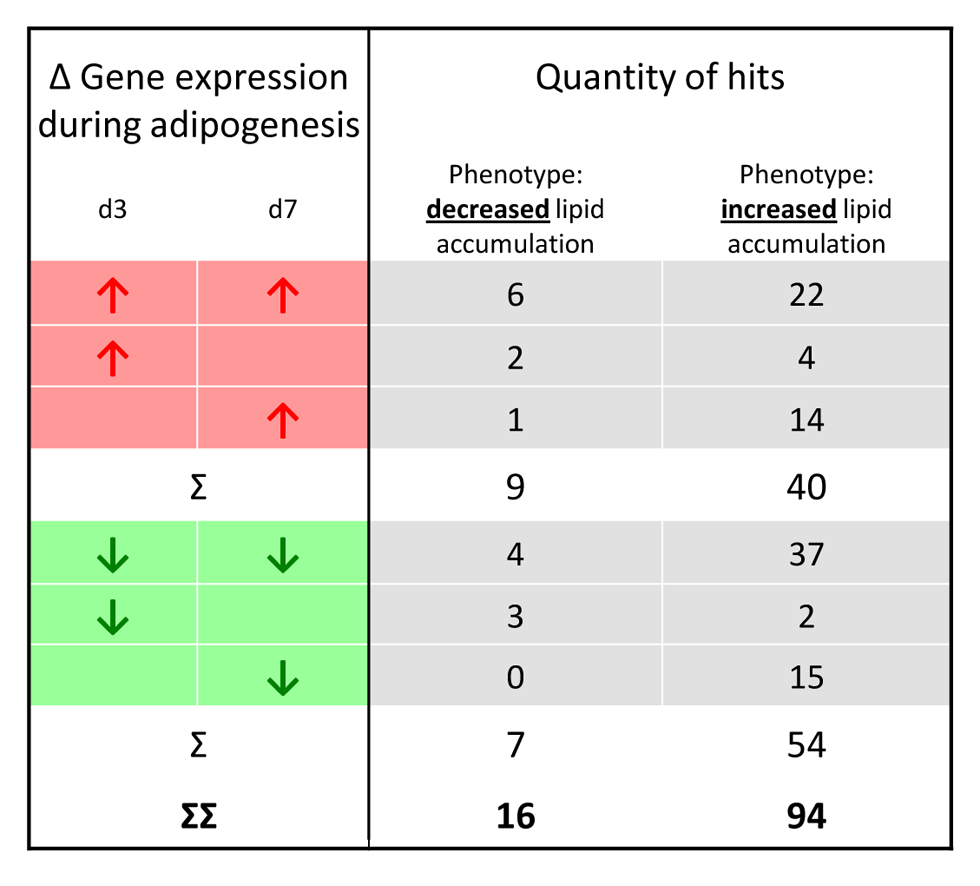

Supplement: Figure S6 — Messenger RNA expression during adipogenesis of hits validated in the secondary screen. Messenger RNA levels were determined by microarray analysis at different time points during adipogenesis (day 0, day 3 and day 7). Depicted is the number of hits displaying an increased (↑) or decreased (↓) gene expression level compared with preadipocytes (fold change ≥2 or ≤−2; p≤0.01). (TIF) [file pone.0031193.s006.tif]
