# Supplementary material for: Identification of New Genes Involved in Human Adipogenesis and Fat Storage
Source: PLoS One. 2012 Feb 27;7(2):e31193. doi: 10.1371/journal.pone.0031193 (PMC3287999; doi:10.1371/journal.pone.0031193)
Supplement: Text S1 — Material and methods for experiments provided in the “supporting information” section. A detailed description is given for: Cell culture and siRNA transfection, qRT-PCR, Immunoblotting, reverse transcription PCR and agarose gel electrophoresis, neutral lipid accumulation, DNA staining, microarray analysis and determination of the lipid correction factors. (DOC) [file pone.0031193.s007.doc]

**Text S1** Supporting Information: Materials and Methods.

**Cell culture and siRNA transfection**

For qRT-PCR and Western blot experiments, siRNA transfection was carried out manually in 6-well format. The following siRNAs (Ambion) were used: *PPARγ*-siRNAs (IDs: 5636, 5731, 5821)*, EPHB4*-siRNAs (IDs: 533, 103322, 103406), *PSKH1*-siRNAs (IDs: 1476, 1570, 1660), *ERBB2*-siRNAs (IDs: 540, 42836, 103546), DNAH7-siRNAs (IDs: 118335, 118337), DNAH8-siRNAs (IDs: 118303, 118305), DNAH17-siRNAs (IDs: 219404, 219403), DNAH9-siRNA (ID: 118306) and DNAI2-siRNA (ID: 118338). The AllStars Negative Control siRNA (Qiagen) was used as non-targeting control. All siRNAs were utilized in a final concentration of 60 nM. SiRNA was diluted (150 pmol/siRNA) in OPTI-MEM I and GlutaMAX I (Gibco BRL) to a final volume of 250 µl. The transfection reagent Lipofectamine 2000 (5 µl, Invitrogen) was diluted with OptiMEM I and GlutaMAX I to a final volume of 250 µl, incubated for at least 5 min, combined with the siRNAs and gently mixed. After 20 min of incubation, 500 µl of the transfection reagent-siRNA complex were transferred to 6 well-plates already containing adherent subcutaneous human preadipocytes (225,000 cells/well in 2 ml culture medium). Cells were incubated at 37°C and 5% CO_2_ for 3 days. After that period of time, adipogenesis was induced for a maximum of 3 days using differentiation medium (see main text).

**qRT-PCR**

To determine gene knock-down on mRNA level, the qRT-PCR technique was used. After transfection, subcutaneous human preadipocytes were cultivated for 3 days with the transfection reagent-siRNA complex. In the following, cells were harvested (preadipocytes) or adipogenesis was induced. Total RNA was then isolated using TRIzol® (Invitrogen) following the manufacturer’s protocol. After reverse transcription with the High Capacity cDNA Reverse Transcription Kit (Applied Biosystems) according to the supplier’s instructions, samples were analyzed by Real-Time TaqMan®-PCR using the 7900HT Fast-Real-Time PCR System (Applied Biosystems).

FAM labelled primers for qRT-PCR (Applied Biosystems) were as follows: Inventoried TaqMan Assays for *GAPDH* Hs99999905_m1), *ERBB2* (Hs01001580_m1), *PSKH1* (Hs00544094_m1), *EPHB4* (Hs00174752_m1), *PPARγ* (Hs00234592_m1) and for the axonemal dyneins *DNAH7, DNAH8, DNAH17* (Assay IDs: see main text), *DNAH9* (Hs00242096_m1) and *DNAI2* (Hs01001544_m1). Using the fast mode, the PCR was performed as recommended by the supplier. Data were analyzed utilizing the Sequence detector version 2.3 software supplied with the 7900HT Fast-Real-Time PCR System (Applied Biosystems). Quantification was achieved using the 2^-∆∆Ct^ method which calculates the relative change in gene expression of the target normalized to an endogenous reference (*GAPDH*).

**Immunoblotting**

For immunoblotting analysis, cells were transfected for 3 days with siRNA. Within the next 3 days adipogenesis was induced using differentiation medium. To obtain cell extracts, cells were washed with 1x phosphate buffered saline (PBS), detached from the culture dish by addition of 0.25% trypsin-EDTA (Gibco BRL) and pelletized (10 min at 250 x g). Cell pellets were resuspended in Laemmli buffer (Bio-Rad, Munich, Germany) containing 5% β-mercaptoethanol (65 µl/1x10^6^ cells). Next, lysates were homogenized with the QIAshredder (Qiagen) and heated for 3 min at 95°C. Lysates (20 µl) were separated using a 10% SDS polyacrylamide gel. Proteins were then transferred to a nitrocellulose membrane (Bio-Rad). After that, membranes were incubated with a primary antibody against GAPDH (dilution: 1:5,000, MA1-16783, Affinity BioReagents, Golden, CO, USA) as a loading control and a primary antibody against PPARγ (dilution: 1:170; #07-466, Millipore, Schwalbach, Germany). Membranes were then incubated with secondary antibodies labeled with IRDye680 (1:20,000) or IRDye800 (1:10,000), respectively (Li-Cor Biosciences, Bad Homburg, Germany). Results were determined using the Odyssey Infrared Imager (Li-Cor Biosciences) with the Odyssey Application Software v. 3.0. The PPARγ signal was normalized to the corresponding GAPDH signal.

**Reverse transcription-PCR and agarose gel electrophoresis**

To clarify if DNAI2 and DNAH9 transcripts are truly present in (pre)adipocytes we performed a PCR analysis using cDNA from DNAI2 knock-down cells and DNAH9-siRNA transfected cells respectively (see above). The PCR reaction was performed with the Thermoprime Plus DNA Polymerase System (ABgene) following the manufacturer’s protocol (cycle no.: 38x). We used two independent primer-sets for each gene (Eurofins mwg Operon, Ebersberg, Germany): DNAI2 (NM_023036); Primer set #1: Left Primer (5’ → 3’): gcagacgccataaagctgac (20); Right Primer (5’ → 3’): tccacttcttcatccccttc (20); Amplicon (110 nt). Primer set #2: Left Primer (5’ → 3’): tggctgctacaatggacaga (20); Right Primer (5’ → 3’): ctggactcaatggtggatagc (21), Amplicon (81 nt). DNAH9 (NM_004662); Primer set #1: Left Primer (5’ → 3’): agctggagctcggcttaaa (19); Right Primer (5’ → 3’): gggcattctgtaagttctcca (21); Amplicon (66 nt). Primer set #2: Left Primer (5’ → 3’): tccattaagatcaccaatgagc (22), Right Primer (5’ → 3’): acacatctccagagtgtcctga (22), Amplicon (93 nt). After amplification, DNA was separated by agarose gel electrophoresis. The DNA was visualized in the 5% gel by addition of ethidium bromide. The DNA size marker GeneRulerTM Ultra Low Range DNA Ladder (Fermentas, St. Leon-Rot, Germany) was used to identify the size of the amplified nucleic acid. Results were analyzed utilizing the AlphaView version 3.2.2 software supplied with the FluorChem FC2 Imager (Cell Biosciences, Santa Clara, California, USA).

**Neutral lipid accumulation**

For analysis of lipid accumulation, siRNA transfection was performed according to the primary screen in the 96-well format, but without the use of a pipetting robot. Following knock-down and induction of differentiation, cells were cultured for 7 days in 96-well plates. For lipid staining (without cell nuclei staining), cells were washed with 200 µl/well 1x PBS. Next, 200 µl/well 1x PBS were added to the cells. In the following, 5 µl AdipoRed^TM^ (Lonza) were added to each well. After incubation for 30 min at 37°C and 5% CO_2_, fluorescence (ex 485/9, em 572/20) was determined in a microplate reader infinite® M200 (Tecan).

**DNA staining**

To determine the cell number directly (by counting) in each cavity of a 96-well plate and to correlate the cell number with Hoechst staining, a propidium iodide staining was carried out. Cells were washed with 1x PBS and 200 µl of a solution consisting of 8.3 µM propidium iodide (Invitrogen) and 20% (v/v) ethanol in 1x PBS were added per well. After 1 h incubation, computer-assisted cell nuclei counting was performed with a fluorescence microscope Axiovert 200M (Zeiss, Jena, Germany) employing the software Zeiss WPA KS 400 Version 3.0 (Zeiss).

For Hoechst 33342 staining, cells were washed with 200 µl/well 1x PBS. Next, 200 µl/well 1x PBS containing 5 µg/ml Hoechst 33342 (Invitrogen) were added to the cells. After incubation for 30 min at 37°C and 5% CO_2_, fluorescence signal (ex 330/80, em 470/40) were determined using a microplate reader Synergy 4 (BioTek Instruments GmbH).

**Determination of the lipid correction factors**

To correct lipid values of the primary screen and the validation experiments for changes in cell number, the Hoechst fluorescence and the signal obtained by measurement of lipids were correlated. To determine the lipid correction factor, a combined lipid and DNA staining was performed 7 days after induction of differentiation using different cell numbers (2,500 – 15,000 cells in each cavity of a 96-well plate). All operational steps of Hoechst 33342 and lipid staining corresponded to those of the primary screen. The slope of the regression line indicates the lipid data correction factor. The lipid data correction factor was determined separately for two cell populations isolated from two different donors.

**Microarray analysis**

To investigate gene expression changes during adipogenesis of genes identified in our screen, microarray analysis were performed. The RNA preparation as well as the microarray analysis were carried out as described in the main text.
